# Allometrically scaling tissue forces drive pathological foreign-body responses to implants via *Rac2*-activated myeloid cells

**DOI:** 10.1038/s41551-023-01091-5

**Published:** 2023-09-25

**Authors:** Jagannath Padmanabhan, Kellen Chen, Dharshan Sivaraj, Dominic Henn, Britta A. Kuehlmann, Hudson C. Kussie, Eric T. Zhao, Anum Kahn, Clark A. Bonham, Teruyuki Dohi, Thomas C. Beck, Artem A. Trotsyuk, Zachary A. Stern-Buchbinder, Peter A. Than, Hadi S. Hosseini, Janos A. Barrera, Noah J. Magbual, Melissa C. Leeolou, Katharina S. Fischer, Seth S. Tigchelaar, John Q. Lin, David P. Perrault, Mimi R. Borrelli, Sun Hyung Kwon, Zeshaan N. Maan, James C. Y. Dunn, Rahim Nazerali, Michael Januszyk, Lukas Prantl, Geoffrey C. Gurtner

**Affiliations:** 1 Division of Plastic and Reconstructive Surgery, Department of Surgery, Stanford University School of Medicine, Stanford, CA USA; 2 Department of Surgery, University of Arizona College of Medicine, Tucson, AZ USA; 3 Department of Plastic Surgery, University of Texas Southwestern Medical Center, Dallas, TX USA; 4 Department of Plastic and Reconstructive Surgery, University Hospital Regensburg, Regensburg, Germany; 5 Cell Sciences Imaging Facility (CSIF), Beckman Center, Stanford University, Stanford, CA USA; 6 Division of Pediatric Surgery, Department of Surgery, Stanford University School of Medicine, Stanford, CA USA

**Keywords:** Translational research, Molecular medicine

## Abstract

Small animals do not replicate the severity of the human foreign-body response (FBR) to implants. Here we show that the FBR can be driven by forces generated at the implant surface that, owing to allometric scaling, increase exponentially with body size. We found that the human FBR is mediated by immune-cell-specific *RAC2* mechanotransduction signalling, independently of the chemistry and mechanical properties of the implant, and that a pathological FBR that is human-like at the molecular, cellular and tissue levels can be induced in mice via the application of human-tissue-scale forces through a vibrating silicone implant. FBRs to such elevated extrinsic forces in the mice were also mediated by the activation of *Rac2* signalling in a subpopulation of mechanoresponsive myeloid cells, which could be substantially reduced via the pharmacological or genetic inhibition of *Rac2*. Our findings provide an explanation for the stark differences in FBRs observed in small animals and humans, and have implications for the design and safety of implantable devices.

## Abstract (web summary)

Elevated extrinsic tissue-scale forces can drive pathological foreign-body responses to implants, mediated by the *Rac2*-mediated activation of a subpopulation of mechanoresponsive myeloid cells.

## Main

Biomedical implants have revolutionized modern medicine by improving the survival and quality of life for millions of patients worldwide. Over 70 million devices, including breast implants, pacemakers and orthopaedic prostheses, are implanted globally each year and are associated with more than US$100 billion in annual expenditure^1^. Chronic inflammation around implanted devices leads to reduced biocompatibility and results in the development of a long-term foreign-body response (FBR). In clinical practice, the longevity of biomedical implants is limited by a pathological FBR, frequently leading to implant failure and eventual rejection^2^. Nearly 90% of all implant failures in commonly used medical devices are associated with the FBR, and up to 30% of all implantable devices will undergo failure during their lifetime^3–5^. As advances in materials science and electronics continue to shape the design of increasingly sophisticated biomedical devices, modifying the underlying host inflammatory response to these biomaterials remains the final frontier in developing truly bio-integrative medical devices.

The FBR begins as a wound-healing-like response to the local tissue trauma that occurs during the initial surgical implantation of a device. Shortly thereafter, the FBR begins a transition towards a long-term response state, in which a fibrous capsule forms around the implant, leading to device malfunction and to the distortion of surrounding tissue^2,6^. The current prevailing hypothesis is that the FBR is primarily a reaction of the local host tissue to the chemical and mechanical surface properties of the implanted material^7–10^. Accordingly, recent research has focused on new chemistries to identify rare, ‘superbiocompatible’ materials, such as zwitterionic hydrogels and triazole-containing alginates, that appear to reduce the FBR^7,11,12^. Additional studies have also explored the potentially important contributions of surface topography, finding that modifying the roughness (micrometre-level surface architecture) could suppress the FBR and fibrosis^10^. Similarly, strategies for modulating the mechanical properties of biomaterials have also been developed and have shown that soft materials can reduce fibrosis, although they do not reduce inflammation^9^. Although these developments have improved our understanding of the FBR, they all have substantial limitations. For example, hydrogels and other soft materials have a low range of elastic moduli (1–100 kPa) and hence cannot be used for biomedical devices that need to provide structural support (such as bone and orthopaedic implants) or for devices that interact with relatively stiffer tissues (such as pacemakers and neurostimulators).

Our incomplete understanding of the FBR is exacerbated by the inability of standard animal models to recapitulate the sustained inflammatory response and severe fibrotic reaction associated with implant failure in humans^13^. Although the molecular machinery responsible for inflammation and fibrosis is highly conserved across species^14,15^, studies have shown that small animal models, such as mice, generate a relatively mild FBR to implantable materials compared with larger organisms^16–18^, such as humans, which limits their clinical relevance. A key feature that differentiates humans from mice is their body size, with humans being several orders of magnitude larger^19,20^. Well-established allometric-scaling principles dictate that tissue-scale forces and, thus, tissue mechanical stress increase exponentially with an increase in body size^19–22^. We have previously shown that increasing the mechanical tissue stress in murine skin during healing promotes the ability of murine models to mimic human-like skin fibrosis^23^. It seems probable that changes in the tissue mechanical environment could also play a role in observed inter-species differences in the FBR, yet the underlying mechanisms have not been examined.

In this study, we report a comprehensive investigation of the FBR in human tissue samples and murine models and determine how changes in the tissue mechanical environment may affect the development of the FBR. Using this knowledge, we then manipulate extrinsic tissue forces using a newly developed murine model to recapitulate the human FBR at histological and transcriptomic levels. In both humans and mice, we identified a unique subpopulation of mechanoresponsive myeloid cells mediated by *RAC2* signalling that specifically respond to changes in tissue forces during the FBR. We also show that the pharmacological and genetic inhibition of these cell populations can prevent the development of a pathological FBR.

## FBRs in humans show similar levels of fibrosis across implant types

To understand the importance of material properties on the human FBR, we analysed fibrotic capsules from a diverse array of biomedical implants. We collected human fibrous-capsule tissue samples from silicone-based breast implants, titanium-based pacemakers, neurostimulators and mixed-alloy-based orthopaedic implants. Because each type of implant was made up of different material chemistries with different mechanical properties, we hypothesized that the resulting fibrotic capsule surrounding each type of implant would vary in fibrotic severity.

**Fig. 1:**
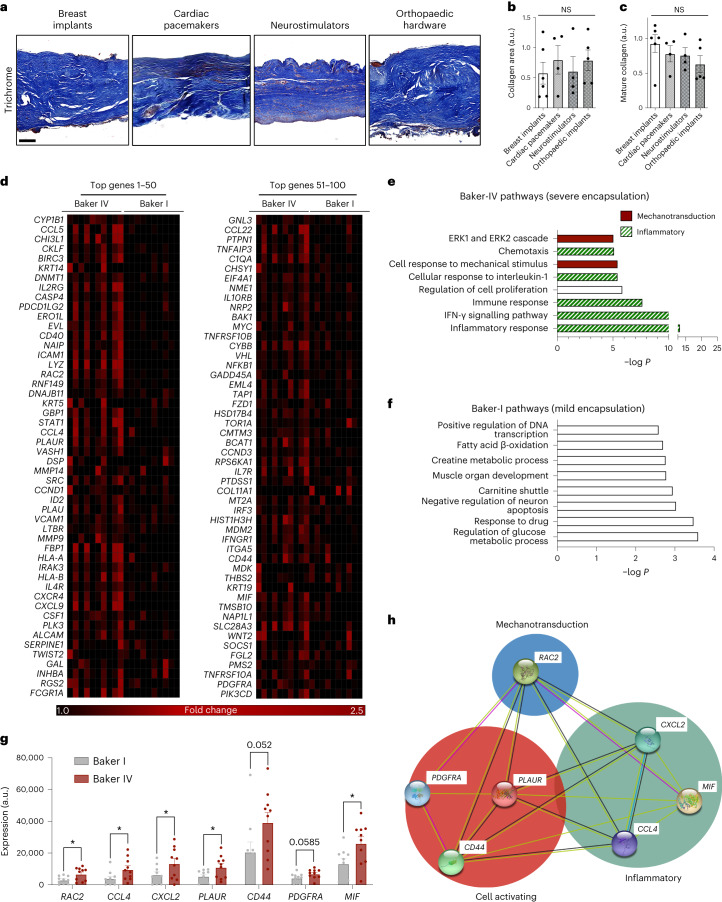
Pathological FBR in humans is mediated by *RAC2* mechanotransduction signalling, regardless of implant properties, and is associated with increased mechanical signalling. **a**, Trichrome staining of fibrotic capsules from the fibrous capsule formed around silicone-based breast implants, titanium-based pacemakers and stainless-steel-based orthopaedic implants are all similar to one another. Implant located at the bottom of each image. Scale bar, 200 µm. **b**,**c**, Quantification of collagen (**b**) and mature collagen (**c**) shows no significant differences between the different types of human implant. For breast implants, *n* = 6 independent capsules; cardiac pacemakers, *n* = 4 independent capsules; neurostimulators, *n* = 4 independent capsules; and orthopaedic hardware, *n* = 5 independent capsules. **d**, Heat map of the top-100 genes upregulated in Baker-IV versus Baker-I breast implants, organized in decreasing order of *P* value. **e**,**f**, Pathways significantly upregulated in Baker-IV (**e**) or Baker-I (**f**) samples analysed using DAVID. Selected pathways highlighted in red are mechanotransduction pathways and those highlighted in green are inflammatory pathways. **g**, Selected cell-activating and inflammatory genes upregulated in Baker-IV (red) (*n* = 10 independent capsules) versus Baker-I (grey) capsules (*n* = 10 independent capsules; *RAC2*, *P = 0.241; *PLAUR*, *P = 0.0388; *CXCR4*, *P = 0.0332; *CD44,* *P* = 0.052; *PDGFRA*, *P* = 0.0585; *MIF*, *P = 0.0211). **h**, STRING analysis showing that *RAC2* is a central mechanotransduction mediator of both cell-activating and inflammatory signalling genes that were all upregulated in Baker-IV specimens. STRING analysed interactions between the different genes based on experimental evidence and predicted interactions. Statistical comparisons for **b** and **c** were made by using a one-way ANOVA with Tukey’s multiple comparisons tests; statistical comparisons for **g** were made using a two-tailed *t*-test comparing Baker-IV with Baker-I samples for each gene. Each data point represents an independent capsule from a different patient. All data represent mean ± s.e.m. Representative images are shown across all experiments. NS, not statistically significant. Source data

**Extended Data Fig. 1:**
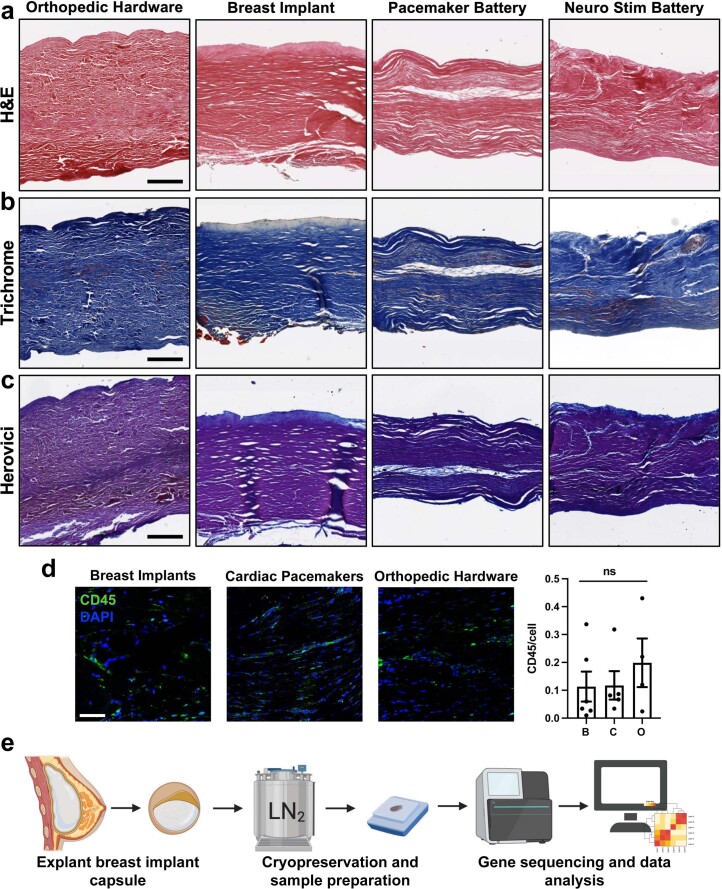
Severe pathological FBR in humans is characterized by similar fibrotic encapsulation, regardless of implant properties. (a) Hematoxylin and Eosin, (b) Trichrome, and (c) Herovici staining of fibrotic capsules from the fibrous capsule formed around silicone-based breast implants, titanium-based pacemakers, and stainless steel-based orthopedic implants are all similar to each other on the tissue architectural level. Scale bar = 200 µm. (d) Immunostaining for CD45 signaling in human implant FBR capsules. Quantification of percent area positive for CD45 in each capsule. Scale bar = 50 µm. Representative images are shown from similar images across n = 5 independent capsules per group. *p < 0.05. Statistical comparisons were made by using a one-way analysis of variance (ANOVA) with Tukey’s multiple comparisons tests. Data is presented as mean ± SEM. B = breast implants, C = cardiac pacemakers, O = orthopedic hardware. (e) Schematic showing the experimental methodology followed; FBR capsules from Baker I and Baker IV breast implants were subject to molecular analyses using a commercially available biomarker panel (HTG Molecular). A total of 10 Baker I specimen and 10 Baker IV specimen were used in this study. Source data

We found that the FBR surrounding each implant was strikingly similar in tissue architecture (Fig. 1a and Extended Data Fig. 1). Specifically, all FBR capsules analysed were predominantly composed of mature type-I collagen with highly organized and aligned fibres, characteristic of severely fibrotic scar tissue resulting from elevated tissue mechanical forces (Fig. 1b,c and Extended Data Fig. 1a–c)^24^. These implants also consisted of relatively similar inflammatory infiltrates (Extended Data Fig. 1d). Overall, these implants all had different material chemistries with different mechanical properties, yet they all generated similar levels of FBR development. Thus, we postulated that the implants’ material properties were insufficient to explain the mechanisms that underlie the human FBR across the range of materials we examined.

## A pathological human FBR is characterized by *RAC2* signalling

To explore other previously unidentified variables that may be involved in the FBR, we analysed implant capsules from identical biomedical breast implants that still generated different severities of FBR in human patients^25^. Fibrosis around breast implants is conventionally classified using the Baker system, where Baker I is the least severe and represents cases with minimal clinically observable implant capsule contracture, whereas Baker IV represents the most severe (pathological) cases that show a sustained inflammatory reaction, pronounced fibrotic contracture and pain. As relatively few patients with mild (Baker I) capsular fibrosis routinely undergo surgical revision and tissue collection, this group represented a limiting factor in our overall data collection. We analysed mRNA isolated from human tissue specimens of both mild (non-pathological) and severe (pathological) FBR observed in humans around implants made of the exact same silicone material using a next-generation-sequencing-based quantitative assay against a biomarker panel (HTG Molecular), which consisted of more than 2,500 known biomarkers for inflammation and fibrosis (Fig. 1d and Extended Data Fig. 1e). We displayed the top-100 genes that were significantly (*P* < 0.05) upregulated in the Baker-IV breast implant capsules compared with the Baker-I capsules in heat maps (Fig. 1d) and then used the Database for Annotation, Visualization and Integrated Discovery (DAVID) pathway analysis to identify the top pathways upregulated in the severe Baker-IV capsules compared with the Baker-I capsules (Fig. 1e,f)^26^. We observed that the top genes upregulated in Baker-IV implants were critically involved in mechanical signalling pathways, including ‘cellular response to mechanical stimulus’ and ‘positive regulation of ERK1 and ERK2 cascade’ (Fig. 1e). Baker-IV implant capsules also showed significant upregulation of ‘inflammatory signalling related to chemotaxis’, ‘cellular response to interleukin-1’ and ‘immune response pathways’ (Fig. 1e). In contrast, the milder Baker-I implant capsules showed only a modest upregulation of pathways related to homoeostatic processes, such as glucose and fat metabolism (Fig. 1f).

As previous research has shown that elevated mechanical forces subsequently activate inflammatory and other cell-signalling processes^27–29^, we postulated that the upregulation of mechanoresponsive signalling genes may also drive cellular responses towards Baker-IV inflammation, fibrosis and pathologically severe FBR. We found that *RAC2*, a haematopoietic-specific Rho-GTPase inflammatory mechanotransduction marker, was significantly (*P* < 0.05) upregulated in the Baker-IV implant specimens (Fig. 1d–g). RAC2 is a signal-transduction molecule that mediates the recruitment and activation of immune cells and that has been shown to be activated by mechanical forces^30,31^. Baker-IV capsules also showed a significantly (*P* < 0.05) increased expression of *CCL4*, which has been shown to be mechanically activated, and mediates classic fibrosis through the activation of haematopoietic cells^29,32,33^. With respect to inflammatory pathways, Baker-IV capsules significantly upregulated *CXCL2* (*P* < 0.05), *PLAUR* (*P* < 0.05) and *MIF* (*P* < 0.05). Of these, *CXCL2* has been shown to contribute to the recruitment and activation of myeloid cells, including neutrophils and macrophages^34–36^. Similarly, *MIF* and *PLAUR* have also been identified as critical mediators of inflammation in haematopoietic cells, leading to fibrosis^37,38^.

Using STRING (Search Tool for the Retrieval of Interacting Genes/Proteins), a pathway analysis tool that predicts gene–gene interactions^39^, we found that *RAC2* could play a centralized and pivotal role as a mechanotransducer that guides the expression of all other aforementioned top genes to drive both cell-activating and inflammatory pathways (Fig. 1h). In addition, *RAC2* guided the activation of *CD44* (*P* < 0.05; Fig. 1g), which has previously been shown to increase the responsiveness of the extracellular matrix to mechanical stimulation and to be crucial for the activation of downstream mechanical effectors, such as *SRC* and *MYC*, which directly contribute to macrophage-mediated inflammation^40–42^.

As both Baker-I and Baker-IV implants are made of the same material (silicone) with identical material chemistry and mechanical properties, these results strongly suggested that pathological (Baker IV) FBR in humans may be mediated by *RAC2* mechanotransduction signalling, independent of implant material properties. As RAC2 is a haemopoietic-specific Rho-GTPase^30,31^, we hypothesized that mechanical forces may mediate immune-cell-specific mechanotransduction to generate pathologically severe FBR. We then sought to test this hypothesis in a murine model.

## Standard murine FBR models generate low mechanical stress

To further study the importance of mechanical forces on the development of FBR, we decided to use a murine model of FBR. Unfortunately, previous studies have shown that mice generate a relatively mild FBR to implantable materials compared with large organisms^16–18^, leading to a dearth of development for clinically relevant therapies for FBR. Because of the dramatic difference in mechanical signalling observed between severe (pathological) and mild (non-pathological) FBR in humans, we hypothesized that increased mechanical signalling owing to allometrically scaling tissue-scale forces might also explain differences in FBR severity between mice and humans. In the context of biomedical implants, cells are believed to mediate mechanotransduction signalling primarily due to the implant material properties. For example, cells on stiffer substrates have been found to exhibit higher mechanical signalling compared with cells cultured on softer substrates^43,44^. However, as organisms evolve and grow larger, fundamental allometric-scaling principles dictate that mice have inherently exponentially lower levels of extrinsic tissue forces compared with humans because of the 10^4^-fold difference in organism size^19–22^. These drastic differences in the surrounding host tissue properties would drastically affect the mechanical environment surrounding the implant. Thus, we must consider both the implant materials’ properties and surrounding tissue properties to quantify the local mechanical stress at the implant–tissue interface.

**Fig. 2:**
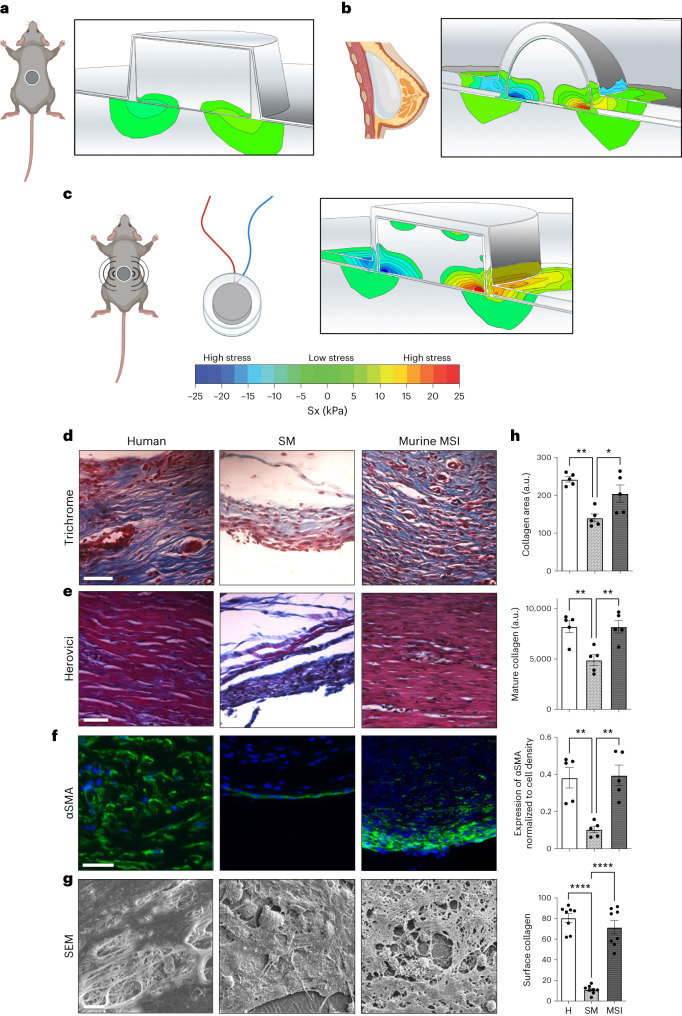
Altering extrinsic tissue-scale forces using MSIs produces a human-like FBR capsule architecture in mice. **a**,**b**, FEM of murine (**a**) and human (**b**) implants showing that human implants are subject to 100-fold higher mechanical stress than murine implants. **c**, Schematic and picture of the MSI model. FEM confirming that MSIs recreate human levels of mechanical stress in the mouse. Sx refers to the mechanical stress in the *x* direction. **d**, Trichrome staining of FBR capsules in the human implant capsules, SM model and the MSI murine model. Scale bar, 50 µm. **e**, Herovici staining showing mature (red) and immature (blue) collagen. Scale bar, 50 µm. **f**, Immunostaining for αSMA, a marker for myofibroblasts. Scale bar, 50 µm. Implant located at the bottom of each image. **g**, SEM imaging of the surface of the capsules. Scale bar, 10 μm. **h**, Top: quantification of percent area positive for collagen in each capsule (far right; *n* = 5 biological replicates for each group; *P = 0.0261, **P = 0.0012). Second from top: quantification of mature collagen deposition in the FBR tissue (far right; *n* = 5 independent capsules per group; **P = 0.0044). Third from top: quantification of αSMA normalized to cell density using image analyses in each capsule (*n* = 5 biological replicates for each group; **P = 0.0028). Bottom: quantification of surface collagen percent area associated with each capsule (*n* = 8 biological replicates for each group; ****P < 0.0001). Statistical comparisons were made by using a one-way ANOVA with Tukey’s multiple comparisons tests. Each data point represents an independent capsule from a different patient or mouse. All data represent mean ± s.e.m. Representative images are shown across all experiments. H, human. Source data

**Extended Data Fig. 2:**
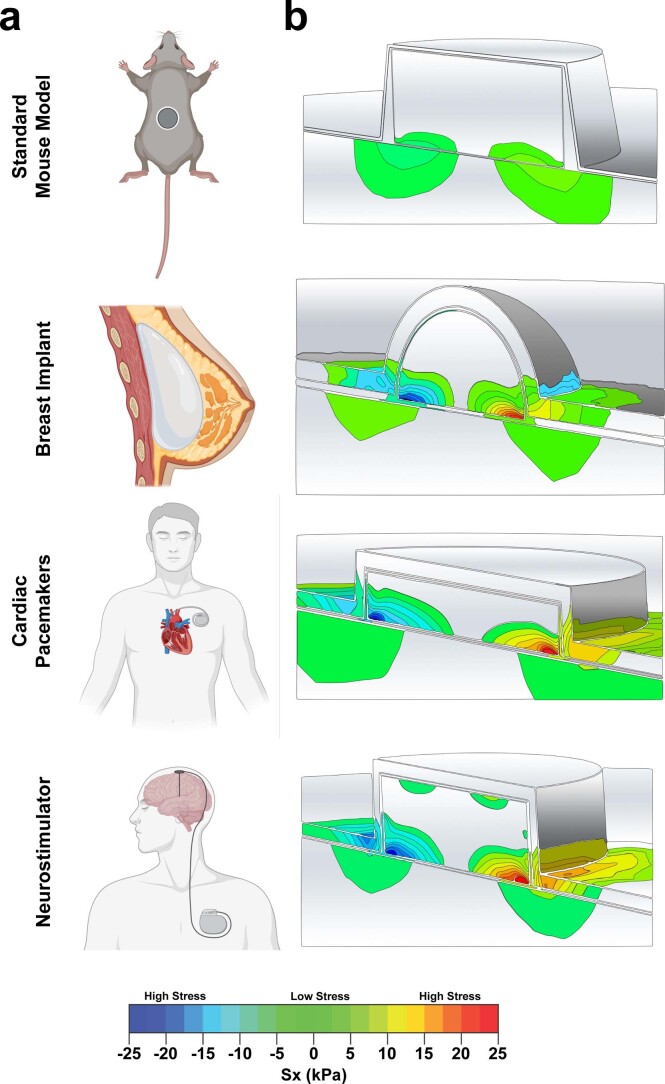
All human implants experience about 100-fold increased mechanical stress compared to standard murine implants. (a) Schematic of standard murine implants and and three commonly used human implants: breast implants, pacemakers, and neurostimulator batteries. (b) FE modeling of standard murine models of FBR reveals minimal mechanical stress at the implant-tissue interface. FE modeling reveals high mechanical stress ( ~ 100-fold higher) around human implants including breast implants, pacemakers and neurostimulator batteries.

To investigate how differences in murine and human extrinsic tissue-scale forces play a role in the development of FBR, we modelled the local mechanical-stress patterns at the implant–tissue interface in mice and humans using finite element modelling (FEM; Abaqus v.2017; SIMULIA). Using a combination of factors, including the murine or human surrounding tissue properties and the material properties of the implants themselves (Methods; Supplementary Tables 1–3 and Supplementary Fig. 1), we predicted that the maximal stress surrounding a standard silicone murine implant was 0.2 kPa, compared with over 20 kPa surrounding human silicone breast implants (Fig. 2a,b and Extended Data Fig. 2). As both implants were made of the same material (silicone), this 100-fold increase in mechanical stress in humans was largely due to the allometric-scaling differences between humans and mice in both tissue size and tissue mechanical properties, potentially explaining why standard murine (SM) preclinical models generate a much lower FBR (Fig. 2a, Supplementary Fig. 1 and Extended Data Fig. 2). Correspondingly, we found that humans generated an FBR with significantly increased amounts of collagen and mature collagen compared with SM models (*P* < 0.05; Fig. 2d–h). Human implant capsules were also characterized by significantly increased myofibroblast activation compared with SM implants (*P* < 0.05; Fig. 2f). Analysing the surface of implants using scanning electron microscopy (SEM), we also observed that human implants were covered by a highly fibrous collagen network, which was not observed on the SM implants (*P* < 0.05; Fig. 2g,h).

Variations in implant geometry and implant material stiffness resulted in only minimal changes to the predicted mechanical stress at the implant–tissue interface in our finite element models. In humans, both silicone-based breast implants and much stiffer titanium-based implants, such as pacemakers and neurostimulators, generated similar mechanical-stress profiles in the range of 20–23 kPa surrounding the implants (Extended Data Fig. 2). These similarities in stress profiles could explain the similar fibrotic encapsulation observed around these different types of human implant (Fig. 1a–c). Overall, by controlling for all other factors including implant material properties, we found that the large differences in murine and human extrinsic tissue mechanical forces significantly contributed to drastically different mechanical-stress profiles surrounding biomedical implants.

## Mechanically stimulating implants induce a human-like FBR

To investigate the importance of these tissue forces, we developed a murine model that artificially imposes human-like levels of increased tissue mechanical forces surrounding the implant, independently of implant size and chemistry. Specifically, we developed an implantable silicone device encapsulating a small motor (Fig. 2c and Supplementary Fig. 2a), which could be induced to produce intermittent in situ implant vibration. As vibration is a mechanical force, these mechanical forces from the implant would intermittently increase the mechanical loading of the surrounding tissue to human levels. After iterating through combinations of vibration frequency and amplitude in our finite element model, we determined that 3 V batteries with an amplitude of 1.38 G and a frequency of 203 Hz would artificially increase the extrinsic tissue-scale forces from the surrounding host tissue to generate a 100-fold increase in mechanical stress at the implant–tissue interface (24.1 kPa), similar to that surrounding human implants (Fig. 2c). The development of these mechanically stimulating implants (MSIs) in mice provided a unique system with which to examine the effect of increased extrinsic tissue-scale forces alone on the subsequent FBR.

When compared with standard implants, MSIs developed FBR capsules with significantly more collagen deposition in the capsule and on the implant surface, increased collagen maturity and upregulated myofibroblast activation (*P* < 0.05; Fig. 2d–h). In accordance, MSIs developed FBR capsules that were nearly identical to human FBR capsules across all these metrics (Fig. 2d–h). These findings showed that by artificially inducing high levels of mechanical stress around murine implants to imitate the mechanical environment in humans, a human-like FBR capsule architecture can be recreated in mice, which is markedly different than that observed in SM implants^13,45^. Both the MSI and standard implants were made of the same material (silicone) and had the same geometry.

**Extended Data Fig. 3:**
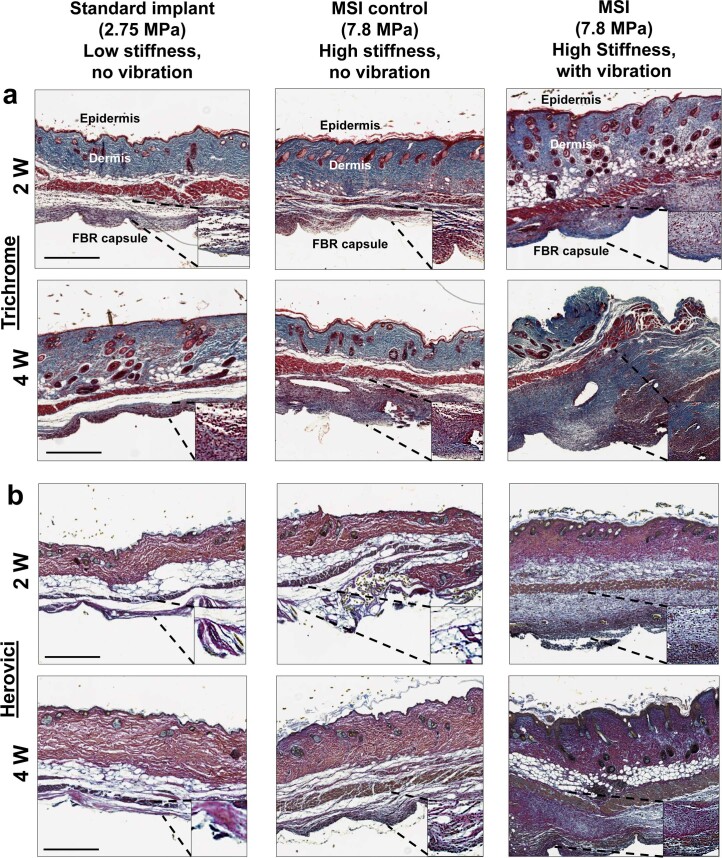
Increased tissue-scale forces result in increased fibrosis around implants in mice, independently of implant chemistry or mechanical properties. (a,b) Trichrome staining and Herovici staining of FBR capsules formed around standard murine implants with low stiffness, standard implants with high stiffness, and MSIs reveals that MSI-model produces a more robust scar tissue, with increased collagen and mature collagen. Representative images are shown from similar images across n = 5 independent capsules per group. Scale bar = 500 μm.

**Extended Data Fig. 4:**
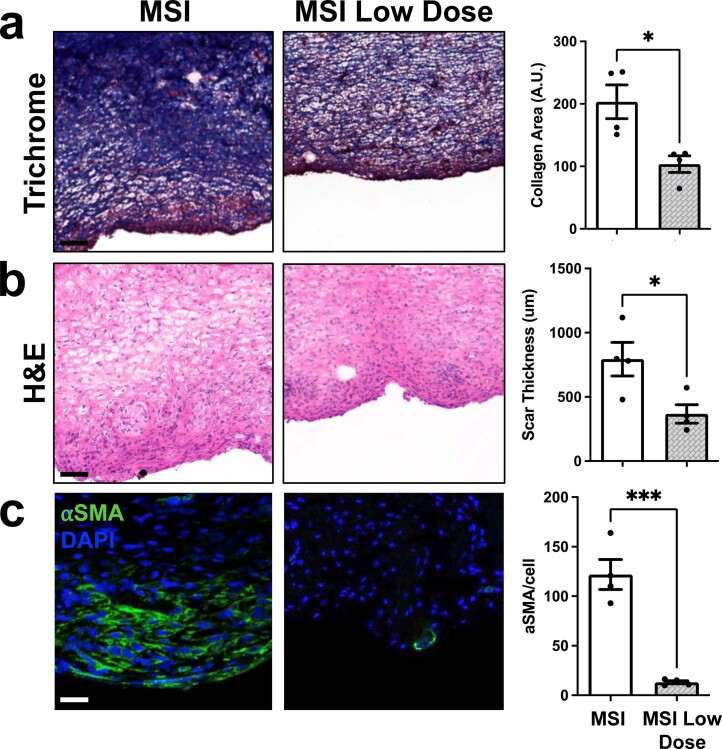
Lower levels of mechanical stress at the implant-tissue interface result in a diminished FBR. (a) Trichrome staining of low-dose MSI stimulated FBR capsules. Scale bar = 50 µm. Quantification of percent area positive for collagen in each capsule (n = 4 for each group. *p = 0.0161). (b) H&E staining of FBR capsules. Quantification of average capsule thickness (n = 4 for each group. *p = 0.0286). (c) Immunostaining for aSMA signaling in FBR capsules (n = 4 independent capsules per group. ***p = 0.0004). Scale bar = 50 µm. All statistical comparisons were made by using two-tailed t tests. All data is presented as mean ± SEM. Source data

To control for any differences resulting from the presence of the inactivated coin motor, we performed additional experiments to compare the FBR between MSIs with the motor on and off (Extended Data Fig. 3). We found that the MSIs without the motor activated were also unable to generate a human-like highly fibrotic capsule (Extended Data Fig. 3), confirming that the human-like FBR observed with the motor activated MSIs (producing vibration) was due to mechanical loading of the surrounding tissue. Furthermore, we found that modulating the amount of mechanical activation with a lower duration of stimulation (lower dose) was also able to titrate the amount of subsequent FBR (Extended Data Fig. 4). Both MSIs and standard implants required surgical incisions and placement of a same-sized silicone disc. As both groups of mice received the same wound injury and implantation of the same-sized silicone device, the only variable that was altered was that of mechanical stimulation. Thus, we show that increased extrinsic mechanical forces by the surrounding tissue results in a human-like highly fibrotic capsule, independent of both material chemistry and mechanical properties (Fig. 2 and Extended Data Fig. 3). Although elevated mechanical tissue forces have been previously linked to increased inflammation and fibrosis in the context of wound healing, we show this phenomenon in the context of the FBR to biomedical implants^46^. Taken together, these results show that manipulating a third independent variable, the extrinsic tissue-scale forces (which can increase either allometrically in humans or artificially with vibration in mice), can drive the biology of the FBR, independent of implant properties.

## Mechanically induced *Rac2* signalling drives pathological FBR

**Fig. 3:**
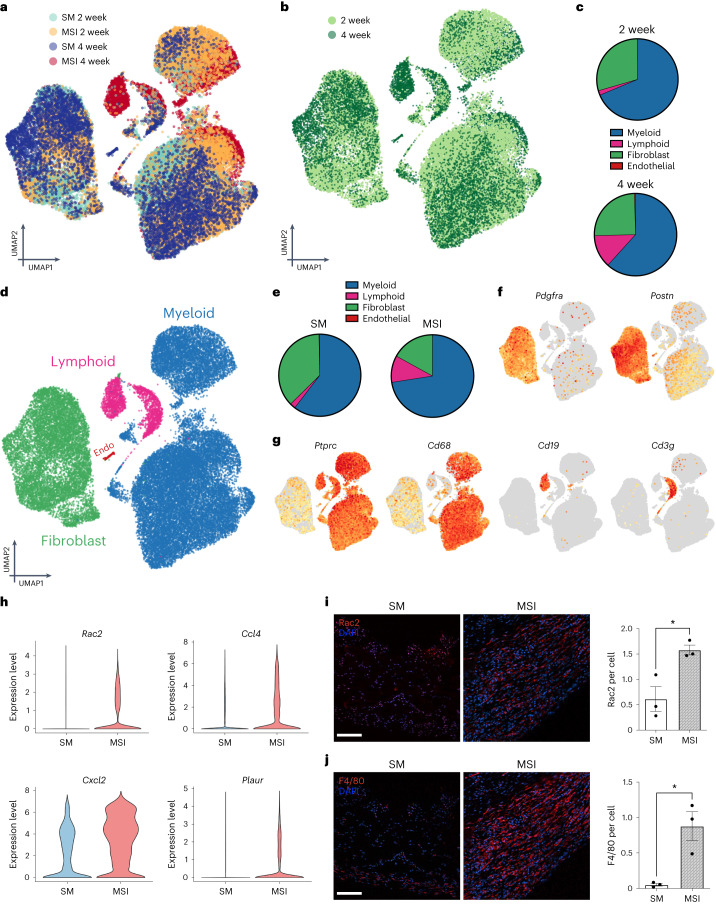
MSIs generate a sustained inflammatory response at the implant–tissue interface. **a**,**b**, UMAP plots of all cells from murine FBR capsules classified by sample type (**a**) and time points (**b**). A total of 36,827 cells were analysed. **c**, Relative proportion of myeloid, lymphoid fibroblast, and endothelial cells in 2-week and 4-week capsules. **d**, UMAP plots coloured by cell type. **e**, Relative proportion of myeloid, lymphoid, fibroblast, and endothelial cells in SM implants and MSI capsules. Myeloid cells were the most abundant cell type in both capsules and were particularly enriched with mechanical stimulation. **f**,**g**, Gene expression of fibroblast-defining genes (**f**) and immune cell-defining genes (**g**) projected onto UMAP embeddings. Grey, no gene expression; light orange, low gene expression; bright orange, high gene expression. **h**, Violin plots of MSI capsules compared with SM implant capsules. **i**,**j**, CODEX immunofluorescence staining of Rac2 (**i**) and (**j**) F4/80. The implant is located at the bottom of each image (*n* = 3 independent capsules per group; Rac2, *P = 0.0212; F4/80, *P = 0.0154). Scale bar, 50 μm. Statistical comparisons for **i** and **j** were made by using a two-tailed *t*-test. Data are presented as mean ± s.e.m. Endo, endothelial. Source data

To examine the cellular response through which extrinsic tissue-scale forces alter the FBR, we analysed the cells surrounding the murine MSI and standard implants using single-cell RNA sequencing (scRNA-seq). We analysed a total of 36,827 cells from both early-stage and late-stage capsules from both standard implants and MSIs (Fig. 3a–c). These time points were chosen due to observable differences in the tissue architecture of the capsules as early as 2 weeks post-implantation (early stage), with the MSI capsules progressing to reach a stable human-like tissue architecture at about 4 weeks post-implantation (late stage) (Fig. 2 and Extended Data Fig. 3). Automated cell identification with SingleR revealed two major cell types that populated the FBR capsules in both standard implants and MSIs: immune cells (myeloid and lymphoid) and fibroblasts (Fig. 3d,e)^47^. Fibroblasts were defined by the cell-specific markers *Pdgfra* and *Postn* (Fig. 3f)^48–51^. Immune cells were defined by the cell-specific markers *Ptprc* (haematopoietic marker), *Cd68* (myeloid cells), *Cd19* (lymphoid B cells) and *Cd3g* (lymphoid T cells) (Fig. 3g)^52–56^. Myeloid cells, including monocytes, macrophages, dendritic cells and granulocytes, were the most abundant cell type in both the standard implant and MSI capsules (Fig. 3d,e), and mechanical stimulus directly increased myeloid cell proportions in the MSI model (Fig. 3e).

**Extended Data Fig. 5:**
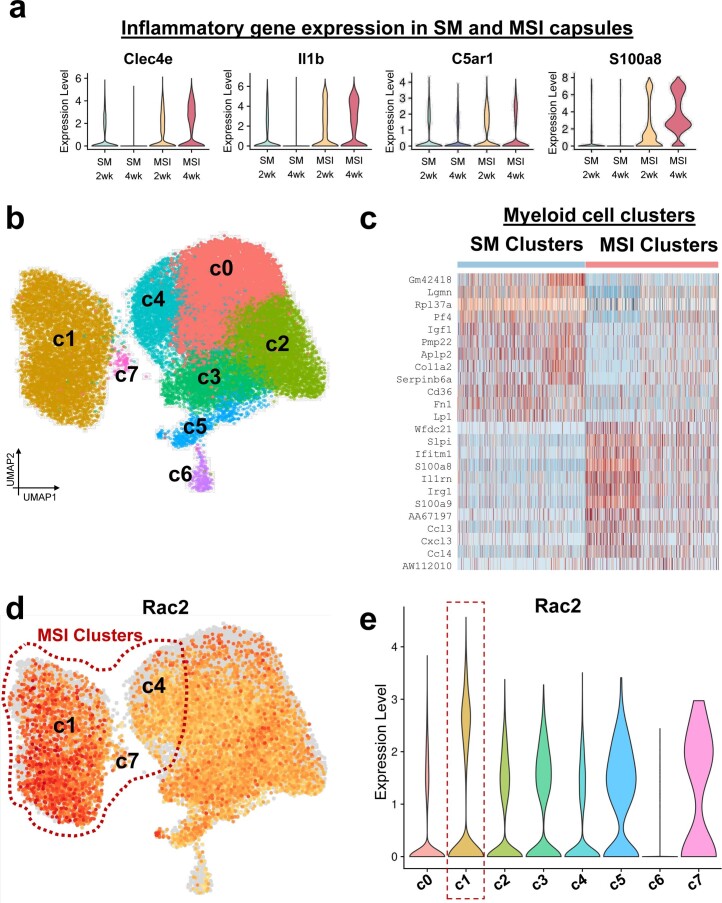
scRNA-seq of cells from SM and MSI implant capsules. (a) Violin plots showing expression of inflammatory genes in standard murine (SM) implants and MSI capsules. (b) UMAP of myeloid cells colored by cluster. (c) Heatmap of differentially regulated genes in myeloid cells. MSI myeloid cells upregulated inflammatory markers. (d) FeaturePlot of Rac2 marker. (e) Violin plots of Rac2 expression in myeloid clusters.

Analysis of the differential gene expression between cells isolated from standard implant capsules and MSI capsules showed significant differences in the activation of *Rac2* and associated inflammatory markers between the two groups. MSI cells showed robust activation of *Rac2* signalling in contrast to standard implant cells, which showed relatively minor activation of *Rac2* (Fig. 3h). In addition, MSI cells showed activation of top markers related to Baker-IV expression in our human data (Fig. 1g), such as *Ccl4*, *Cxcl2* and *Plaur*. Similarly, MSI cells showed robust activation of inflammatory markers, such as *Il1b*, *Clec4b* and *C5ar1* (Extended Data Fig. 5a)^57–59^. Standard implants initially expressed a modest activation of these inflammatory markers, which subsided at later time points, whereas MSI capsules showed a robust early activation of these markers, which continued to increase in later time points (Extended Data Fig. 5a). Thus, our murine MSI model recapitulated the overall upregulation of *Rac2* signalling and inflammation induced by mechanical stimulus that was observed in the transcriptomic profiles of human Baker-IV severely fibrotic capsule tissue (Fig. 1).

To confirm these findings at the protein level, we then performed co-detection by indexing (CODEX) immunofluorescence staining for an array of markers specifically related to these cell types and genes related to human Baker IV (Fig. 3i,j). To start, we first confirmed the overall expression of RAC2 and F4/80 (macrophage) in our tissue samples. We observed that MSI tissue indeed expressed a significant upregulation of RAC2 (*P* < 0.05) expression (Fig. 3i) and F4/80 macrophage (*P* < 0.05) numbers (Fig. 3j). After confirming that our MSI model recapitulates the RAC2-mediated mechanotransduction environment observed in humans, we then proceeded to characterize the critical cell types that drive the FBR, starting with myeloid cells.

## *Rac2* immune signalling drives the Baker-IV FBR

**Fig. 4:**
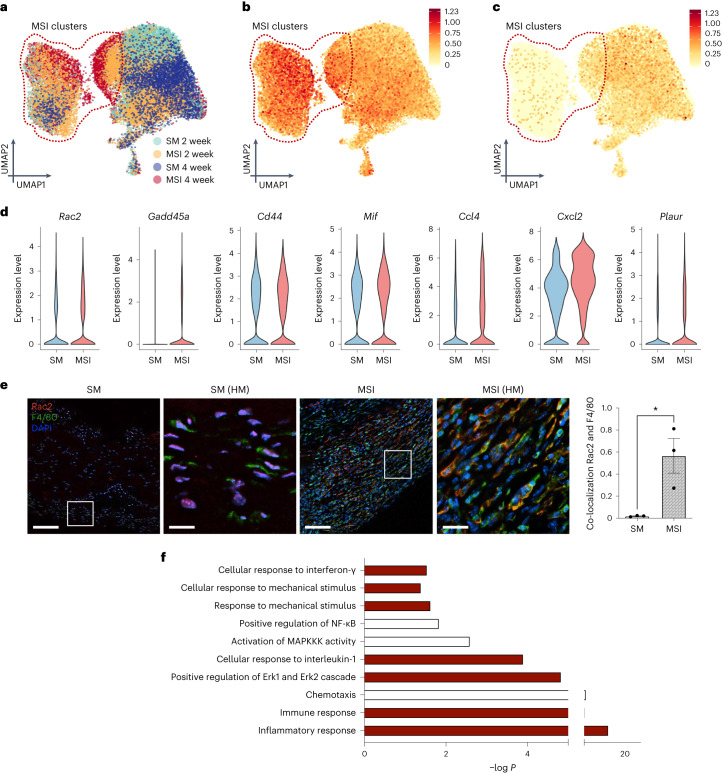
Increased extrinsic tissue-scale forces activate *Rac2* signalling in myeloid cells, which drives a Baker-IV fibrotic phenotype in mice. **a**, UMAP plot of myeloid cells in SM and MSI implant capsules. Clusters 1, 4 and 7 (red dotted line) are highly enriched in MSI capsules. **b**,**c**, FeaturePlot of top averaged Baker-IV markers (Fig. 1d), including key mechanotransduction and inflammatory chemokine signalling pathways (**b**) and Baker-I markers (**c**). Color bar denotes the amount of gene expression. **d**, Violin plots of Baker-IV markers differentially upregulated in the MSI clusters (arbitrary units). **e**, CODEX immunofluorescence staining of co-localized pixels of Rac2 and F4/80 (*n* = 3 independent capsules per group, *P = 0.0254). White box denotes high magnification (HM) image area. Scale bar for SM and MSI, 50 μm. Scale bar for HM images, 10 μm. Statistical comparisons for **e** were made by using a two-tailed *t*-test. Data are presented as mean ± s.e.m. **f**, Selected pathways significantly upregulated in murine MSI samples analysed using DAVID. Pathways highlighted in red are also upregulated in Baker-IV human specimens. Source data

We separately analysed each major cell type (that is, myeloid cells, lymphoid cells and fibroblasts) to determine cell-type-specific transcriptional shifts induced by increased levels of extrinsic tissue-scale forces. We first focused our analysis on myeloid cells because they made up the majority of the capsules and seemed to directly increase with mechanical stimulus (Fig. 3d,e). Myeloid cells clustered in 8 distinct subpopulations (clusters c0–c7; Extended Data Fig. 5b). Of these myeloid cell clusters, clusters c1, c4 and c7 were specifically enriched in MSIs (Fig. 4a and Extended Data Fig. 5b).

Next, we examined how the transcriptional profiles of these murine clusters compared with the expression of Baker-IV human biomarkers (Fig. 1), that is, the genes that were highly upregulated in Baker-IV (pathological) human specimens. We determined the combined mean expression of the top-25 Baker-IV biomarkers (Fig. 1d), which included mechanical signalling and downstream inflammatory genes, to create a ‘gene signature’ that represented the transcriptomic profile of pathological, severe human FBR. Plotting the expression of this gene signature, we found that genes related to Baker-IV pathological FBR were highly upregulated in MSI clusters (clusters 1, 4 and 7) compared with expression in standard implant clusters (Fig. 4b). We then created a gene signature of the top-25 Baker-I biomarkers and found that standard implant clusters instead upregulated genes found in the mild human Baker-I capsules (Fig. 4c), which indicated normal homoeostatic processes^60,61^.

In addition, *Rac2* and downstream mechanotransduction genes (for example, *Mif* and *Cd44*) and inflammatory genes (for example, *Ccl4*, *Cxcl2* and *Plaur*) were also differentially upregulated in the MSI clusters, as with the top differential genes of interest observed in human Baker-IV capsules (Fig. 1d,g, Fig. 4d and Extended Data Fig. 5c)^37,62,63^. Specifically, although all myeloid cells expressed some baseline level of *Rac2* expression, MSI-enriched clusters such as cluster 1 expressed higher levels of *Rac2* (Extended Data Fig. 5d,e). To confirm the specific upregulation of *Rac2* in macrophages, we again used our CODEX staining to look at co-localization of Rac2 and F4/80 expression (Fig. 4e). Indeed, our MSI model significantly (*P* < 0.05) increased the co-localization of Rac2 and F4/80, confirming that increasing the local mechanical environment directly stimulated the differentiation of macrophage-specific Rac2 expression.

DAVID pathway analyses further confirmed the strong overlap in the gene expression between MSI cells and human Baker-IV capsules (Fig. 4f). Murine MSI cells from both time points upregulated key mechanotransduction pathways, including ‘positive regulation of Erk1 and Erk2 cascade’ and ‘cellular response to mechanical stimulus’, and inflammatory pathways, such as ‘interferon-γ signalling’, ‘chemotaxis’, ‘immune response’ and ‘inflammatory response’ (Fig. 4f). These pathways all matched the pathways upregulated in human Baker IV capsules (Fig. 1e).

Overall, these myeloid cells expressed a wide array of genes and pathways with marked overlap with human FBR transcription, with mechanical stimulation of immune cells promoting a pathological Baker-IV profile and SM implants promoting a benign Baker-I profile. The differential upregulation of inflammatory markers along with the increased presence of myeloid cells in MSI capsules further showed that the human-like FBR in MSIs is characterized by a mechanically induced and sustained inflammatory response at the implant–tissue interface.

## Elevating tissue forces reproduces cellular FBR properties

**Fig. 5:**
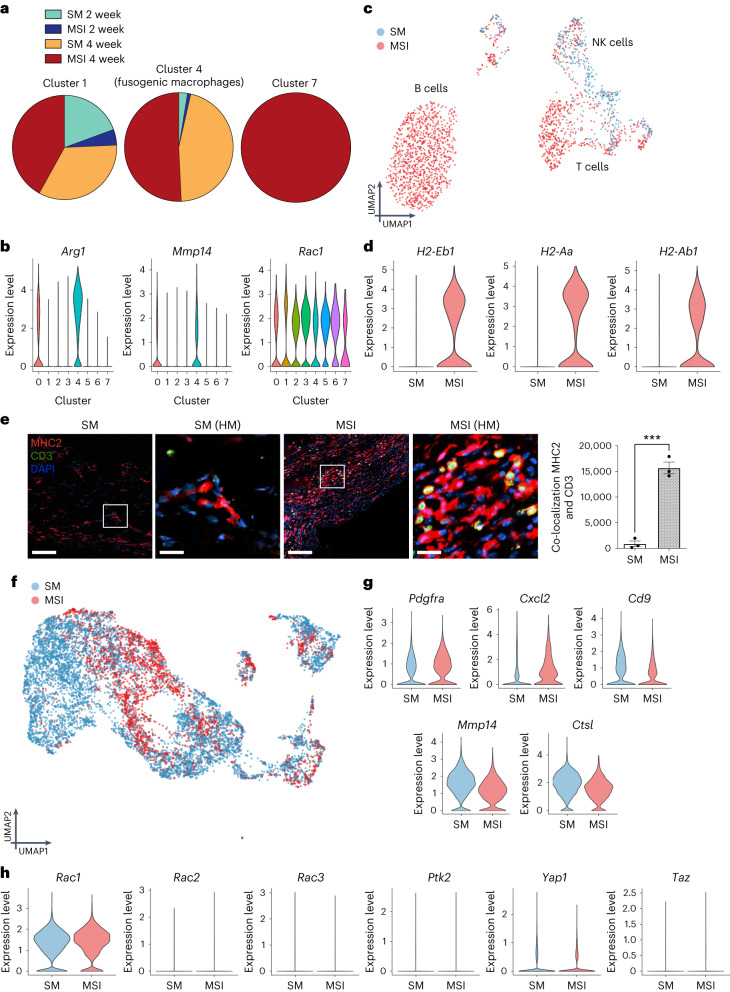
Increasing tissue-scale forces activates fusogenic macrophages, MHC II lymphocytes and myofibroblasts—all classic features of a pathologic FBR. **a**, Relative proportions of cell types in cluster 1, 4 and 7 cells (primarily MSI macrophage clusters). **b,** Violin plots of gene expression (arbitrary units) by cell cluster. Cluster 4 cells upregulate markers for fusogenic macrophages. **c**,**d**, UMAP (**c**) and violin plots (arb. units) (**d**) of lymphocytes from murine FBR capsules. MSI lymphocytes show upregulation of MHC class II signalling. **e**, CODEX immunofluorescence staining of co-localized pixels of MHC2 and CD3 protein (*n* = 3 independent capsules per group, ***P = 0.0003). White box denotes high magnification (HM) image area. Scale bar for SM and MSI, 50 μm. Scale bar for HM images, 10 μm. Statistical comparisons were made by using a two-tailed *t*-test. Data are presented as mean ± s.e.m. **f**,**g**, UMAP (**f**) and violin plots (arb. units) (**g**) of fibroblasts from murine FBR capsules. **h**, Violin plots (arb. units) of mechanotransduction markers in SM and MSI fibroblasts. NK, natural killer. Source data

Fusion of macrophages, leading to the formation of foreign body giant cells, is a hallmark of the classic FBR^6^. Fusogenic macrophages and foreign body giant cells are known to release degradative enzymes, reactive oxygen species and pro-fibrotic factors, which regulate the recruitment, growth and proliferation of fibroblasts. We found that *Arg1*^*+*^ macrophages (cluster 4), which have been previously reported to be fusogenic macrophages, were highly enriched in MSI capsules compared with standard implants (Fig. 5a,b)^64^. It has been reported that *Rac1*-mediated fusion of macrophages requires the expression of *Mmp14* and that *Mmp14*-null cells do not fuse^65^. Cluster 4 macrophages showed a simultaneous upregulation of *Arg1*, *Rac1* and *Mmp14* (Fig. 5b), showing the activation of fusogenic macrophages in MSIs in response to increased tissue-scale forces at the implant–tissue interface. The presence of these fusogenic macrophages was confirmed using SEM of the implant surface in our human biomedical implants (Supplementary Fig. 3).

**Extended Data Fig. 6:**
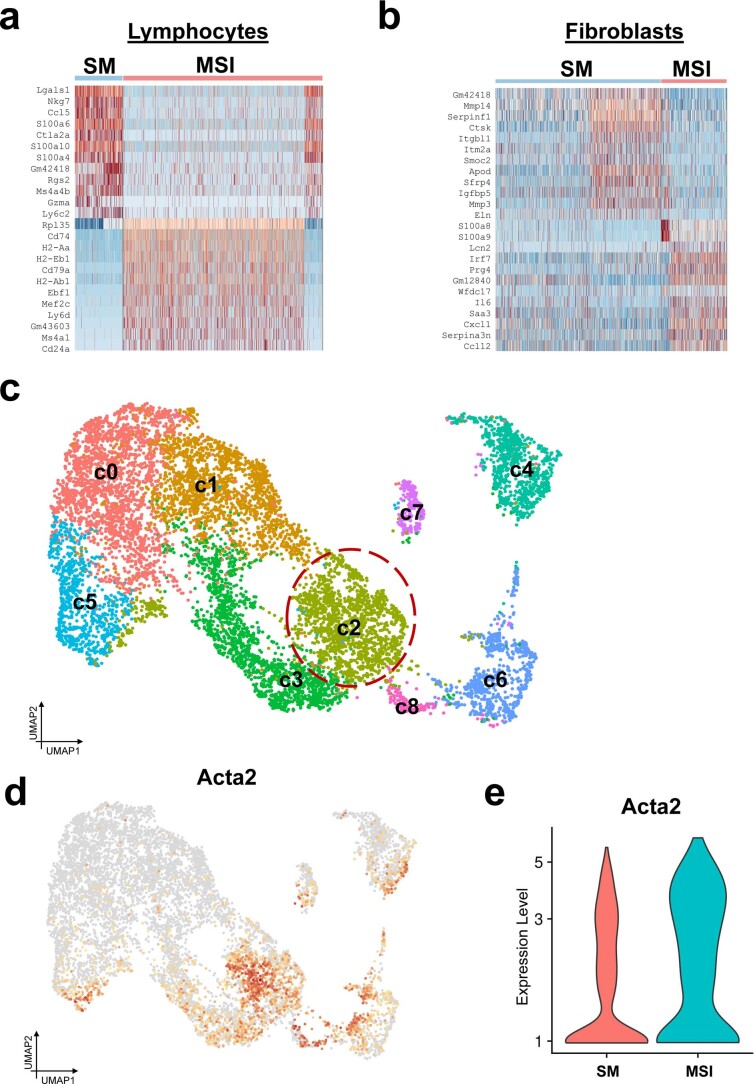
Differentially upregulated fibroblast and lymphoid cell genes. (a) Differentially upregulated genes between lymphocytes cells from the standard murine implants and MSIs. (b) Differentially upregulated genes between fibroblast cells from the standard murine implants and MSIs. (c) UMAP of fibroblasts colored by cluster. (d) Feature Plot of Acta2 marker in fibroblast population. (e) Violin plot of Acta2 expression in SM and MSI myofibroblast cluster.

As macrophage fusion is promoted by activated lymphocytes, we next analysed all lymphocytes in our dataset to identify the lymphoid-specific changes in transcriptional activity induced by mechanical stimulation. MSI lymphocytes showed a preferential upregulation of major histocompatibility complex (MHC) class II FBR components, including *H2-Eb1*, *H2-Aa* and *H2-Aa1* (Fig. 5c,d and Extended Data Fig. 6a). This was further confirmed on the protein level using CODEX to show significantly (*P* < 0.05) upregulated co-localization of MHC class II expression with lymphocytes through CD3 expression (Fig. 5e). This provided evidence that the mechanical activation of lymphocytes may then signal macrophage fusion to contribute to increasing severity of FBR^6,66,67^.

As expected, we also observed the presence of fibroblasts in the implant capsules. Historically, fibroblasts are thought to be critical in the FBR because these cells are responsible for synthesizing collagen that cross-links in the extracellular space and contributes to the formation of a dense, collagen-rich fibrotic capsule around the implant^68^. We observed that several inflammatory cytokines linked to the activation of fibroblasts, such as *Cxcl2*, *Plaur* and *Ccl4*^37,62,63,69,70^, were upregulated in MSI capsules (Fig. 1g and Fig. 4d). We then compared fibroblasts from both implant models (Fig. 5f,g and Extended Data Fig. 6b) and found that fibroblasts in the SM model showed upregulation of proteolysis (*Mmp14* and *Ctsl*) and negative regulation of cell proliferation (*Cd9*), suggesting a resolving phenotype^71–73^. In contrast, MSI fibroblasts showed an upregulation of myofibroblast marker *Pdgfra*, pro-fibrotic cytokines such as *Cxcl2* and downregulation of the anti-proliferation marker *Cd9* (Fig. 5g)^49,71,74^, indicative of a more activated fibroblast phenotype in MSIs. Fibroblasts clustered in several distinct subclusters (Extended Data Fig. 6c), including cluster 2 *Acta2*^*+*^ cells, which we defined as our myofibroblast cluster (Extended Data Fig. 6d). Even within the myofibroblast cluster, we observed increased *Acta2* expression in MSI fibroblasts compared with SM cells (Extended Data Fig. 6e). These results showed that increased myofibroblast differentiation and collagen deposition occurred in MSI capsules, consistent with our protein-level observations of increased myofibroblasts populating the MSI capsules (Fig. 2f).

Fibroblasts from MSI capsules and standard implant capsules showed minimal activation of canonical fibroblast mechanotransduction genes such as *Ptk2*, *Yap1* and *Taz* (Fig. 5h)^21,68^. In addition, fibroblasts showed almost identical levels of *Rac1* activation and minimal activation of *Rac3* (Fig. 5h). As *Rac2* is a haematopoietic-specific marker^75–77^, fibroblasts did not express *Rac2*, further increasing our confidence that immune cells are the primary cell type responsible for the initial *Rac2*-mediated mechanotransduction to drive FBR. As *Rac2* expression is differentially upregulated in FBR capsules and myeloid cells make up the majority of cells in chronic FBR capsules, immune cells serve as a primary mechanosensor in both murine and human FBR. Thus, it appears that although fibroblasts produce collagen to create fibrotic tissue, the activation of fibroblasts is primarily mediated by activating these immune cells in the context of FBR.

## Blocking *Rac2* signalling significantly reduces pathologic FBR

**Fig. 6:**
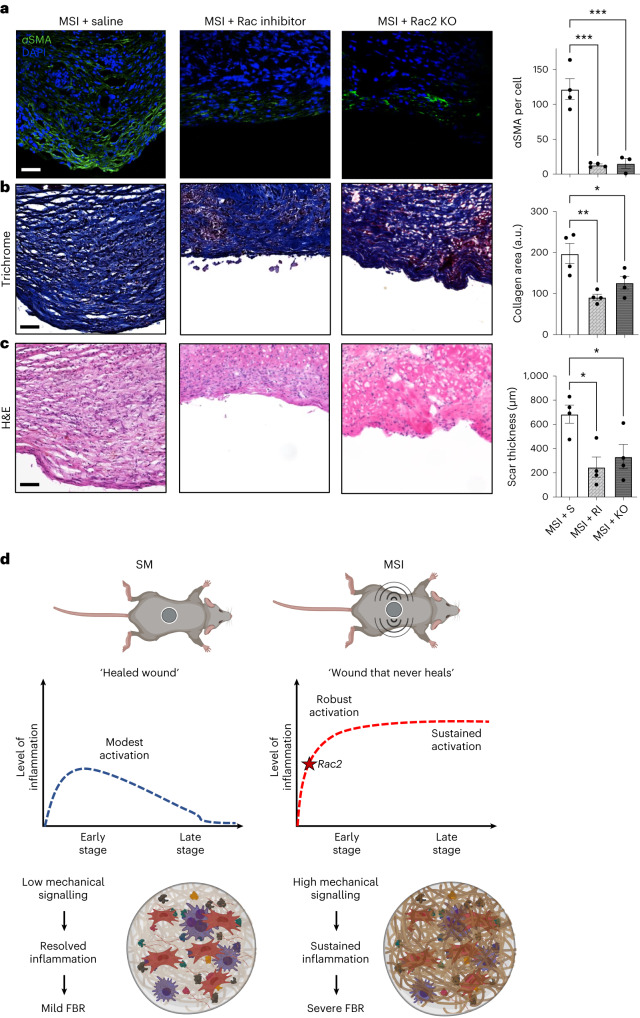
Blocking *Rac2* signalling effectively reverses the human-like FBR induced by increased tissue-scale forces in mice. Comparative analysis of histology sections of FBR capsules from the MSI mouse model either pharmacologically blocked with Rac inhibitor (RI) or genetically blocked in a *Rac2*^−/−^ global KO mouse. **a**, Immunostaining for αSMA signalling in FBR capsules. Quantification of percent area positive for αSMA in each capsule (*n* = 5 biological replicates for each group; ***P = 0.0003). **b**, Trichrome staining of FBR capsules. Quantification of percent area positive for collagen in each capsule (*n* = 5 biological replicates for each group; *P = 0.045, **P = 0.0051). **c**, Haematoxylin and eosin (H&E) staining of FBR capsules. Quantification of average capsule thickness (*n* = 4 biological replicates for each group; *P = 0.0153). Scale bar in **a**–**c**, 50 µm. **d**, In SM implants, there is a modest activation of inflammatory pathways at the early time point, which subsides at the late time point, resulting in minimal FBR. In contrast, in MSI capsules, increased tissue-scale forces lead to the activation of *Rac2* mechanical signalling, which promotes a robust activation of inflammatory markers that is sustained over time, resulting in a human-like pathological FBR. Statistical comparisons were made by using a one-way ANOVA with Tukey’s multiple comparisons tests. Each data point represents an independent capsule from a different mouse. All data represent mean ± s.e.m. Representative images are shown across all experiments. S, saline. Source data

**Extended Data Fig. 7:**
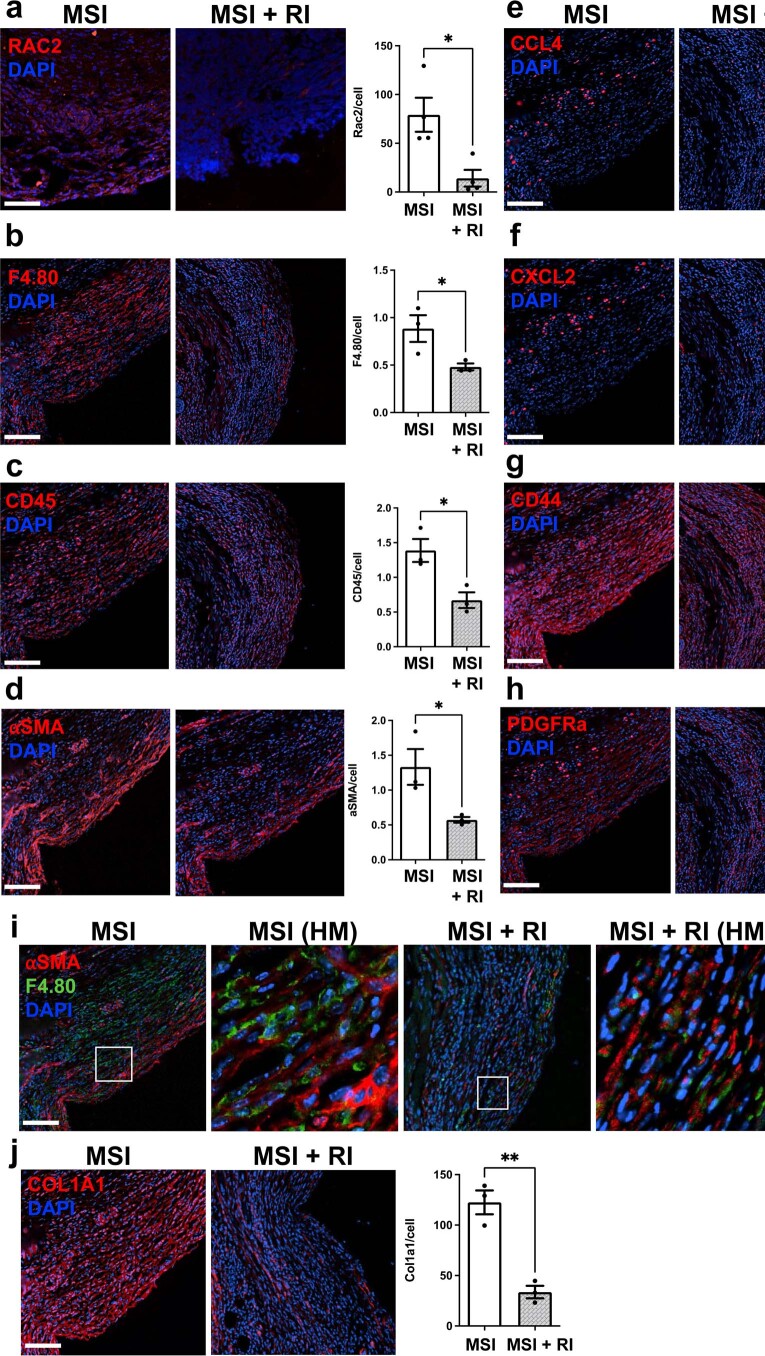
Immunostaining for upregulated genes. (a) Immunostaining for RAC2 signaling in FBR capsules (*p = 0.0155). Scale bar = 50 µm. Quantification of percent area positive for RAC2 in each capsule. (b-d) CODEX immunofluorescent staining for cellular populations of (b) F4/80 (macrophages, *p = 0.0499), (c) CD45 (all immune cells, *p = 0.0231), and (d) aSMA (fibroblasts, *p = 0.0433). (e-h) CODEX immunofluorescent staining for top Baker IV human genes (e) CCL4 (*p = 0.0338), (f) CXCL2 (*p = 0.0231), (g) CD44 (*p = 0.0478), and (h) PDGFRA (*p = 0.0411). (i) CODEX immunofluorescent co-staining for F4/80 (macrophage) and αSMA (fibroblast) co-localization. Quantification of how many aSMA expressing cells also express F4/80. (j) CODEX immunofluorescent staining for collagen marker COL1A1 (**p = .0027). Scale bar = 100 µm). n = 3 independent capsules per group for all groups. All statistical comparisons were made by using two-tailed t tests. All data is presented as mean ± SEM. MSI = mechanically stimulated implant. RI = Rac inhibitor. Source data

Our findings show that mechanical tissue-scale forces activate *Rac2* signalling in immune cells, which drive the classic human pathological FBR. As the extrinsic tissue-scale forces are inherent to the size of the organism and cannot be altered, it would require either pharmacological or transgenic strategies to block the mechanical activation of immune cells in FBR. Thus, we tested the efficacy of pharmacological inhibition using a small molecule Rac inhibitor (EHT 1864 2HCl)^78^ and a transgenic, *Rac2*^−/−^ global knockout (KO) mouse model in reducing FBR in our MSI model (Fig. 6). First, local injection of EHT 1864 2HCl in the MSI model reduced the expression of immune cell-specific *Rac2* in the FBR capsules by about 80%, indicating a significant reduction in the recruitment and activation of mechanoresponsive immune cells (Extended Data Fig. 7a). Next, we observed a significant reduction in the activation of myofibroblasts in MSI capsules either treated with the small molecule inhibitor or in the transgenic mice by about 90% (Fig. 6a). Blocking *Rac2* signalling in mice either pharmacologically or genetically significantly reduced the overall FBR as well, which was specifically shown by decreased capsule thickness and collagen deposition (Fig. 6b,c).

We then confirmed our findings on the protein level using CODEX immunofluorescence staining (Extended Data Fig. 7b–i). First, we observed that use of a Rac inhibitor significantly reduced immune cell populations in our murine MSI capsules using immune cell markers F4/80 (macrophages, *P* < 0.05) (Extended Data Fig. 7b) and all immune cells with CD45 (*P* < 0.05) (Extended Data Fig. 7c). Next, we also confirmed that myofibroblast markers were reduced such as α-smooth muscle actin (αSMA, *P* < 0.05; Extended Data Fig. 7d). In addition, inhibition of Rac expression also significantly reduced the expression of Baker-IV markers shown in Fig. 1e, such as CCL4 (*P* < 0.05; Extended Data Fig. 7e), CXCL2 (*P* < 0.05; Extended Data Fig. 7f) and CD44 (*P* < 0.05; Extended Data Fig. 7g), and another fibroblast marker PDGFRa (*P* < 0.05; Extended Data Fig. 7h), which we showed was upregulated in our MSI model in Fig. 5g. As some recent studies have shown that macrophages could also potentially be αSMA positive^79^, we then found that less than 20% of the αSMA signal is co-localized with F4/80 (Extended Data Fig. 7i), showing that most of the SMA-positive cells are not macrophages and are indeed myofibroblasts. Finally, we found that inhibition of Rac expression significantly reduced the expression of collagen marker COL1A1 (*P* < 0.01) in our murine MSI capsules (Extended Data Fig. 7j).

Taken together, these results show that blocking *Rac2* signalling in immune cells can cause a cascade of downstream effects, including significantly decreased myofibroblast differentiation, reduced downstream collagen production and mitigated FBR capsule formation. In short, by blocking the immune orchestrators of FBR, it is possible to reverse the human-like FBR resulting from increased levels of extrinsic tissue-scale forces in mice.

## Discussion

Previous research has identified implant chemistry and mechanical properties as critical factors in mediating the FBR^7,9,10^. In this work, we have identified a third independent variable—extrinsic tissue-scale forces—playing a central role in the FBR. In humans, extrinsic tissue-scale forces increase exponentially with body size owing to allometric-scaling principles, creating a high-mechanical-stress environment around biomedical implants. In contrast, mice have low extrinsic tissue-scale forces, which leads to a low-mechanical-stress environment and to a minimal FBR. Our experiments show that murine physiological mechanical stress is insufficient to facilitate a human-like FBR. Instead, inducing human levels of mechanical stress in mice can drive the formation of human levels of FBR. These dramatic changes in tissue forces can explain the stark differences in FBR between murine and humans, independent of the chemistry and mechanical properties of the material.

By using an MSI, we artificially increased the extrinsic tissue-scale forces and recapitulated all aspects of the human FBR at both the histological and transcriptional levels. It is important to note that we compared FBR responses with SM implants and MSIs using the same material and with exactly the same implant geometry. By varying only tissue-scale forces, we were able to dramatically alter the molecular and cellular response to recreate a human-like FBR in mice. Our results provide an explanation for the long-standing conundrum in the field regarding the inter-species variability in FBR, despite the fact that the molecular machinery responsible for inflammation and fibrosis is highly conserved among species^14,15^. We show that larger-sized organisms, such as humans, experience higher tissue-scale forces because of allometric scaling that then drive subsequent FBR.

The wound-healing cascade in humans is characterized by sequential inflammatory and fibrotic phases and eventual quiescence^80^. The FBR begins as a wound-healing response to the local tissue damage that occurs during surgical implantation of a device. However, continually elevated levels of extrinsic mechanical forces create a high-mechanical-stress environment surrounding the implant that leads to a sustained inflammatory response. In SM implants with 100-fold-lower mechanical stress than in humans, we observed a modest inflammatory response at the early stage, which subsided by later time points similarly to a ‘healed wound’ (Fig. 6d). In contrast, both human implants and murine MSIs generate a 100-fold-increased stress environment to perpetuate a sustained presence of mechanically activated immune pathways throughout the FBR. Thus, increased tissue-scale forces, shaped by allometric-scaling principles in humans, result in a ‘wound that never heals’, which leads to pathological FBR. In fact, allometrically scaling tissue-scale forces may provide the missing link explaining other key aspects of the pathological FBR, including the activation of fusogenic macrophages, MHC class II lymphocytes and myofibroblasts.

We found that both murine and human pathological FBR capsules mostly comprised immune cells and that extrinsic tissue-scale forces result in the mechanical activation of *Rac2* signalling in a unique population of immune cells, with a gene signature conserved in both mice and humans. Most previous literature has focused on mechanoresponsive fibroblasts as the main cell types contributing to fibrosis, responding to the highly mechanically stressed environment by secreting cytokines and upregulating inflammatory cell recruitment^81,82^. Our findings show a more dynamic interplay between immune cells and fibroblast activity, and suggest that mechanoresponsive immune cells under elevated tissue-scale forces may actually drive and regulate the activation of fibroblasts. As the pathophysiology of FBR develops in humans, immune cells recruited to initially surround the implant may respond to the highly mechanically stressed tissue environment in the surrounding tissue. This activation of pathways such as RAC2 could then trigger the secretion of fibroblast-activating cytokines, such as CCL4 and CXCL2, which then activate fibroblasts to become more contractile and produce excess collagen that contributes to severe FBR. Some recent reports have indeed emphasized the potentially critical role of immune cells in FBR^83,84^, and future work will be performed to interrogate these cellular relationships more carefully. Our findings provide a mechanistic link for the activation of immune cells in the FBR, namely, *Rac2* signalling in immune cells activated by allometrically scaling tissue-scale forces in humans in the FBR capsule.

The allometric scaling of tissue properties cannot be altered because it is inherent to the size of the organism. Hence, biomolecular and pharmacologic strategies will be required to create truly bio-integrative devices. Our findings suggest that the pharmacological inhibition of *Rac2* might serve as an effective therapy for humans receiving biomedical implants to prevent the FBR, to increase the patient’s quality of life and to reduce implant-failure rates. For potential future large-animal and human clinical trials, a range of dosages of inhibitor must be studied, and a translationally relevant drug-delivery method would need to be developed. For example, future biomedical implants could have a coating of the inhibitor, and systemic therapy could also be investigated. Overall, our findings contribute to the better understand of the FBR, provide a mechanistic target to prevent the development of a pathological FBR, and may have substantial implications for the design and safety of implantable medical devices in humans.

## Methods

### Animals

All mice used in this study were housed in the Stanford University Veterinary Service Center and Stanford University animal care guidelines were followed. All procedures were approved by the university’s Administrative Panel on Laboratory Animal Care. C57/BL6 female, wild-type mice (8 weeks old; stock number 000664; Jackson Laboratories) and C57/Bl6.129S6-Rac2^tm1Mddw^/J Rac2 KO female mice (8 weeks old; stock number 004197; Jackson Laboratories) were used in these experiments.

### Human implant capsule specimen

Explanted biomedical devices (breast tissue expanders and implants, neurostimulator batteries, pacemakers and orthopaedic implants) and the surrounding capsular tissue were collected for this study and analysed. Informed consent was obtained from each patient in accordance with the Institutional Review Board at Stanford University (number 41066).

### Human tissue bank and RNA analysis

We used a large tissue bank for human breast implant capsule tissues, located at the University of Regensburg, Germany, which consists of over 710 unique breast tissue samples^25^. As relatively few patients with Baker-I capsules undergo revisionary surgery, our overall sample size was limited by this group. We were able to identify 10 samples of Baker-I capsules in our biobank, and this determined the sample size for this study (*n* = 10 for Baker-I samples; *n* = 10 for Baker-IV samples). The patients were of comparable ages: (1) 40.6 ± 3.9 years at the time of implantation in Baker-I and 35.8 ± 4.40 years in Baker-IV implants, and (2) 50.3 ± 3.0 years at the time of explant in Baker-I and 51.0 ± 4.0 years in Baker-IV implants. The patients had silicone breast implants placed for augmentation for a mean of 10.7 ± 2.8 years in Baker-I and 15.2 ± 4.5 in Baker-IV implants. None of the patients previously had cancer. No other patient-identifying information was recorded. For RNA analysis, 5 µm formalin-fixed paraffin-embedded sections of human samples were lysed, proteinase K digested and analysed by the HTG EdgeSeq Quantitative Nuclease Protection Assay (qNPA) (HTG Molecular Diagnostics) using a biomarker panel (HTG Oncology Biomarker Panel), a 2,549-gene probeset, including markers for inflammation and fibrosis. After EdgeSeq qNPA processing, samples were individually barcoded by polymerase chain reaction and pooled for sequencing. Libraries were sequenced on the Illumina NextSeq platform (Illumina) and data were processed with HTG’s parser software. Approval was given by the local ethic committee in Regensburg (reference number 15-101-0024). Differential expression analysis was performed with the EdgeR package in R (v.3.14.0) with Benjamini–Hochberg correction for multiple hypothesis testing^85^. The 100 most highly ranked genes from this analysis for Baker-I and Baker-IV implants were used to perform gene-set enrichment analysis against pathway databases using the DAVID toolkit as previously described^26^.

### STRING analysis

To study the interaction of *RAC2* with other Baker-IV genes, STRING (a pathway-analysis tool that predicts gene–gene interactions) was used as previously described^39^. The minimum required interaction score was set at 0.200. STRING analysed interactions between the different genes based on experimental evidence and the predicted interactions. The relative positions of nodes and the distances between the different nodes are arbitrary. The gene–gene interactions are colour-coded as follows: pink, for experimentally determined; blue, curated databases; green, gene neighbourhood; red, gene fusion; dark blue, gene co-ocurrence; light green, text mining; black, co-expression; and violet, protein homology.

### Implant fabrication

Standard silicone implants were made of polydimethylsiloxane (PDMS) and fabricated using a Sylgard 184 elastomer base and curing agent as previously described^86^. An elastomer:curing agent ratio of 5:1 was used for the experiments described. All implants were cylindrical in shape with a 1.55 cm diameter and 0.67 ± 0.07 cm height. For MSIs, a pre-fabricated coin motor (Precision Microdevices) was placed in the elastomer solution before curing (Fig. 2c), whereas controls were PDMS alone. To enable in situ vibration of MSIs, the wires from the implant had to be guided through the skin, which required a new surgical technique (Supplementary Fig. 2a). After skin incision and creation of a subcutaneous pocket on the back of the mice, two 20 G cannulae were inserted into the pocket in a cranio-caudal direction. The wires were tunnelled through the pocket and guided through the skin using the cannulae and a modified Seldinger technique, enabling activation of the motor by an external battery. MSIs could then be attached to the external battery for an hour every day during the fibroproliferative phase of FBR (days 4–11), as outlined in Supplementary Fig. 2b. Both MSI and SM implant models required surgical skin incisions and silicone disc (1.55 cm diameter and 0.66 + 0.07 cm height) placement. Longer durations of vibration were not well tolerated by mice. A 3 V power source was chosen in accordance with our FEM to most accurately match the mechanical stress around implants in humans. As a second control, MSI implants were also tested without in situ vibration.

### Implant mechanical testing

The Young’s modulus (*E*_y_) of silicone implants was determined using a custom compressive test method on Instron 5560 as described previously^87^. Each sample had a diameter of 1.55 cm and subjected to a compressive rate of 1 mm s^−1^. *E*_y_ of each implant was calculated by taking the linear slope of the stress–strain curve between 0 and 0.10 compressive strain.

### Implantation experiments

Standard silicone implants and MSIs were implanted in C57/BL6 mice for either 2 weeks or 1 month. A 2 cm incision was made on the dorsum of the mouse and a subcutaneous pocket was created. Control implants were placed in the subcutaneous pocket and the incision was closed using 6-0 nylon suture or staples. For MSIs, the implants were placed into a subcutaneous pocket, similar to the procedure described above, and wires were guided through the skin using a modified Seldinger technique. Batteries (3 V) with an amplitude of 1.38 G and a frequency of 203 Hz were used to mimic human conditions. MSIs were vibrated for 1 hour daily from day 4 post-implantation to day 11 (Supplementary Fig. 2b). This time period was chosen based on previous studies, which showed that increased mechanical stress during this period effectively induces fibrosis^23,46^.

### Computational modelling of mechanical-stress patterns around biomedical implants

Computational finite element models for human and mouse implants were developed using the commercial finite element software Abaqus (v.2017; SIMULIA), using a similar FEM framework as previously described to study mechanical behaviour of soft tissues and investigate deformation and stress patterns in biological tissues^88–90^. Model geometry was based on experimental measurements of skin and fat layers and custom-designed implants for humans and mice^22,91,92^. In the initial configuration, the implants were modelled as a three-dimensional disc (Supplementary Fig. 1). Fat, skin and muscle were represented as layers around the implant. Movement of implants transfers deformation and force to the layers of skin and fat. In all models, the bottom end of the muscle/bone layer was fixed. Tetrahedral elements (C3D4) were used for soft tissue layers, whereas hexagonal elements (C3D8) were used for implants. Mesh refinement confirmed that the chosen mesh size is accurate enough for the purposes of this study.

The simulation was based on the theory of elastic deformation of soft tissue where for each human and mouse model, different material properties were considered for skin, fat and muscle/bone layers based on previous findings (Supplementary Tables 1–3)^22,93,94^. The models contained external loading as static or vibrating forces, where the direction of the applied force was in the horizontal axis in all models. Once the force was applied to the implant, this force moves the three-dimensional geometry of the implant in the direction of the applied load. Due to the defined tie interaction between the implant and tissue layers around it, implant movement applies the stress to the tissue where tissue on both ends of the implant experience negative (compression) and positive (stretch) stresses (Fig. 2). The magnitude of these stresses was proportional to the stiffness and elastic properties of the tissue. The stress experienced by the tissue further triggers mechanotransduction pathways, leading to biological responses. The models contained external loading as static or vibrating forces, where the direction of applied force was in the horizontal axis in all models. For human and mouse static models (Fig. 2a,b), the amplitude of the applied force was calculated from dynamic resting tensions previously reported^23^. For the MSI model (Fig. 2c), a periodic force was defined for vibrating implants as 1.38x the standard gravitational constant (9.8 m/s^2^), so the amplitude of the vibrating force was

$${\left({F}_{\mathrm{static}}\right)}_{\mathrm{human}}=1.38g\approx 13.5\,{\mathrm{N/kg}}.$$

### Histology and trichrome staining

At each time point, the mice were euthanized, and the implants were resected en bloc with the surrounding scar tissue. The implants were removed from the surrounding tissue, and the scar tissue along with the skin was fixed in 4% paraformaldehyde overnight. Samples were then subjected to serial, hour-long ethanol washes followed by three 1.5 hour xylene washes before being embedded in paraffin. Scar tissue and implants from humans were collected from patients and processed within an hour. For analysis of the capsule, paraffin blocks were trimmed and cut into 5-µm-thick paraffin ribbons in a warm water bath before being mounted onto glass slides. Paraffin slides were then stained with trichrome (Sigma-Aldrich) as previously described^23,46^. Imaging was performed on a Leica DM5000 B upright microscope. Image analysis software (ImageJ) was used to quantify collagen staining.

### Herovici’s staining

Herovici’s staining was performed according to manufacturer specifications as described below. Thin histological sections were deparaffinized and immersed in Weigert’s haematoxylin solution for 5 minutes followed by Herovici’s working solution (equal parts of stain solutions A and B) for 2 minutes. Slides were subsequently immersed in 1% acetic acid, followed by dehydration using alcohol and xylene washes. Finally, slides were mounted using mounting media with a coverslip on top. Imaging was performed on a Leica DM5000 B upright microscope. Image analysis software (ImageJ) was used to quantify mature collagen (red colour) staining.

### SEM

Tissue on the surface of implants was fixed using 4% paraformaldehyde. Samples were dehydrated using a series of ethanol washes with increasing concentration from 70% to 100% ethanol for 5 minutes each. The samples were subsequently immersed in hexamethyldisilazane for 15 min and then sputter coated with gold palladium before imaging with SEM. For image analysis, at least eight different SEM images with collagen fibres on the surface of the implant were analysed using ImageJ for each group.

### Immunostaining

Immunohistological staining was performed on paraffin sections as previously described^46,95^. Briefly, heat-based antigen retrieval was followed by blocking with 5% goat serum in PBS. The following primary antibodies were used at a 1:200 dilution (as recommended by the manufacturer) and incubated overnight at 4 °C: anti-αSMA (ab5694; Abcam) or anti-Rac2 (AB_2547156; Fisher Scientific). Incubation of primary antibody-stained specimens with Alexa Fluor 488 secondary antibody (Thermo Fisher Scientific) was performed at a 1:400 dilution for 1 hour at room temperature. Sections were subsequently mounted using Fluoroshield (F6057; Sigma) with 4′,6-diamidino-2-phenylindole (DAPI) to stain cell nuclei. Imaging was performed on a Leica DM5000 B upright microscope. Image analysis was performed using MATLAB^21,44^.

### Single-cell barcoding, library preparation, and scRNA-seq

Implant capsule tissue was explanted from murine MSI experiments and processed for single-cell sequencing as previously described^96,97^. Freshly obtained tissue from the clinic was micro-dissected and digested with collagenase to obtain cellular suspensions for 10× single-cell sequencing (Single Cell 3′ v.2; 10x Genomics) according to the manufacturer’s instructions. Briefly, a mixture of droplet-based single-cell suspensions, partitioning oil and the reverse-transcription master mix were loaded onto a single-cell chip, and reverse transcription was performed on the Chromium controller at 53 °C for 45 min. Complementary DNA was amplified for 12 cycles on a Bio-Rad C1000 Touch thermocycler using SpriSelect beads (Beckman Coulter) and a ratio of SpriSelect reagent volume to sample volume of 0.6. cDNA was analysed on an Agilent Bioanalyzer High Sensitivity DNA chip for qualitative control. cDNA was fragmented using the proprietary fragmentation enzyme blend at 32 °C for 5 min, which was followed by end repair and A-tailing for 30 min at 65 °C. cDNA were double-sided size selected using SpriSelect beads, followed by ligation with sequencing adaptors at 20 °C for 15 min. cDNA amplification was performed using a sample-specific index oligo as primer, and subsequently another round of double-sided size selection using SpriSelect beads was performed. Final libraries were analysed on an Agilent Bioanalyzer High Sensitivity DNA chip for quality control and were sequenced using a HiSeq 500 Illumina platform, aiming for 50,000 reads per cell.

### Data processing, FASTQ generation and read mapping

The Cell Ranger software (v.3.1; 10x Genomics) implementation of mkfastq was used to convert base calls to reads. These reads were then aligned against the mm10 v.3.0.0 genomes using Cell Ranger’s count function (STAR v.2.7.0) with SC2Pv2 chemistry and 5,000 expected cells per sample^98^. Cell barcodes representative of quality cells with at least 200 unique transcripts and less than 10% of their transcriptome of mitochondrial origin were analysed.

### Data normalization and cell subpopulation identification

Raw unique molecular identifiers (UMIs) from each cell barcode were normalized with a scale factor of 10,000 UMIs per cell. The UMI reads were then natural log transformed with a pseudocount of 1 using the R package Seurat (v.3.1.1)^99^. Highly variable genes were identified, and cells were scaled by regression to the fraction of mitochondrial transcripts as previously described^97^. The aggregated data were subsequently evaluated using uniform manifold approximation and projection (UMAP) analysis over the first 15 principal components, and cell annotations were ascribed using SingleR toolkit (v.3.11) against the Immgen and mouse RNA-seq databases.

### Generation of characteristic subpopulation markers and enrichment analysis

Seurat’s native FindMarkers function with a log-fold-change threshold of 0.25 using the Receiver Operating Characteristic (ROC) test was used to generate marker lists for each cluster. The most highly ranked genes from this analysis were used to perform gene-set enrichment analysis against pathway databases for each cluster or subgroup of cells using the DAVID toolkit as previously described^26^.

### Rac inhibition and *Rac2* KO experiments

EHT 1864 2HCl, a potent Rac family GTPase inhibitor, was acquired from Selleckchem. MSIs were implanted in C57/BL6 wild-type mice for 28 days, using the modified Seldinger technique previously described. Mice were injected with EHT 1864 2HCl (10 mg kg^−1^ per day; *n* = 4) or saline (*n* = 4) from day 0 to 26. The method of injection was as follows: mice were manually restrained using the scruff method. A tent of skin directly above the implant was pulled taught to separate the epidermal and dermal layers from the implant and musculature of the animal, creating a subcutaneous capsular space. A needle was inserted into the subcutaneous space and aspirated before drug administration to ensure a clear field. EHT 1864 2HCl was subsequently injected into the subcutaneous capsule space. FBR capsule tissue was explanted on day 28 and processed for histologic analysis.

For the *Rac2* KO experiments, MSIs were implanted in C57/Bl6.129S6 mice. MSIs were implanted in these C57/BL6 wild-type mice for 28 days using the modified Seldinger technique described above. As *Rac2* is exclusively expressed in haematopoietic-derived cells, the transgenic *Rac2*^−/−^ mouse is an immune-cell-specific KO model. FBR capsule tissue was explanted on day 28 and processed for histologic analysis.

### CODEX immunofluorescence staining

DNA oligonucleotide barcodes and conjugation kits (containing all buffers) were obtained from Akoya Biosciences. For conjugations, 50 mg of purified, carrier-free antibodies were used. See the Supplementary Information for details on antibody clones and barcode information. Staining was done according to the Akoya Biosciences staining protocol. Automated image acquisition and fluidics was performed using Akoya’s software driver CODEX Instrument Manager (CIM v.1.29) and the CODEX platform (Akoya Biosciences). Imaging was performed using a Keyence BZ-X810 microscope, fitted with a Nikon CFI Plan Apo l 20×/0.75 objective. Eleven *z* steps were acquired with the pitch set at 1.5 in the BZ-X software. Raw TIFF files were processed using the CODEX Processor v.1.7.0.6 by Akoya Biosciences.

### Statistical analyses

Results are presented as mean ± s.e.m. Standard data analysis was performed using student’s *t*-tests. Analysis of variance (ANOVA) with post hoc Tukey’s test was used for multiple comparisons. Results were considered significant for *P* < 0.05.

### Reporting summary

Further information on research design is available in the Nature Portfolio Reporting Summary linked to this article.

### Supplementary information

Supplementary Information Supplementary tables and figures.

Reporting Summary

Supplementary Data 1 Antibody panel for CODEX run.

Supplementary Data 2 Cell-defining genes.

Supplementary Data 3 Human genes, ordered by *P* value.

Supplementary Data 4 Human genes (raw).

Supplementary Data 5 Myeloid Seurat clusters.

Supplementary Data 6 HTG molecular raw data.

### Source data

Source Data Figs. 1–6 and Extended Data Figs. 1, 4 and 7 Source data.

## Data Availability

The main data supporting the results in this study are available within the article and its Supplementary Information. The scRNA-seq data are available from the NCBI Gene Expression Omnibus via the series accession number GSE227908 (https://www.ncbi.nlm.nih.gov/geo/query/acc.cgi?acc=GSE227908). Source data for the figures are provided with this paper. The raw and analysed datasets generated during the study are available from the corresponding authors on reasonable request.

## Code availability

All custom code is available from the corresponding authors on reasonable request.
