# Supplementary material for: Allometrically scaling tissue forces drive pathological foreign-body responses to implants via Rac2-activated myeloid cells
Source: Nat Biomed Eng. 2023 Sep 25;7(11):1419–36. doi: 10.1038/s41551-023-01091-5 (PMC10651488; doi:10.1038/s41551-023-01091-5)
Supplement: Supplementary file 1 — Supplementary tables and figures. [file 41551_2023_1091_MOESM1_ESM.pdf]

nature biomedical engineering

Article https://doi.org/10.1038/s41551-023-01091-5

Allometrically scaling tissue forces drive pathological foreign-body responses to implants via *Rac2*-activated myeloid cells

In the format provided by the authors and unedited

Supplementary Table 1. Average values for skin and subcutaneous tissue properties used for modelling.

	**Human Model** (*μm*)	**Mouse Model** (*μm*)
**Thickness of skin**	2400	200
**Thickness of fat-top**	500	100
**Thickness of fat-bottom**	500	100
**Thickness of fat-upper side**	500	100
**Thickness of fat-lower side**	1000	200

Supplementary Table 2. Dimensions for human and mouse tissue and implants.

	**Human Model** (*cm*)	**Mouse Model** (*cm*)
**Height of Pacemaker**	0.75	NA
**Radius of Pacemaker**	2.5	NA
**Height of Chest Battery**	1.5	NA
**Radius of Chest Battery**	2.5	NA
**Radius of Breast Implant**	12	NA
**Height of Implant**	NA	0.67
**Radius of Implant**	NA	0.75

Supplementary Table 3. Material properties (Young's Modulus) of human and mouse models.

	**Human Model**	**Mouse Model**
**Skin**	108.19 KPa	13.22 KPa
**Fat**	3.25 KPa	3.5 KPa
**Muscle**	18.6 KPa	8.9 GPa
**Control Implant**	NA	2.75 MPa
**MSI**	NA	7.80 MPa
**Pacemaker**	103 GPa	NA
**Chest Battery**	103 GPa	NA
**Breast Implant**	12 MPa	NA

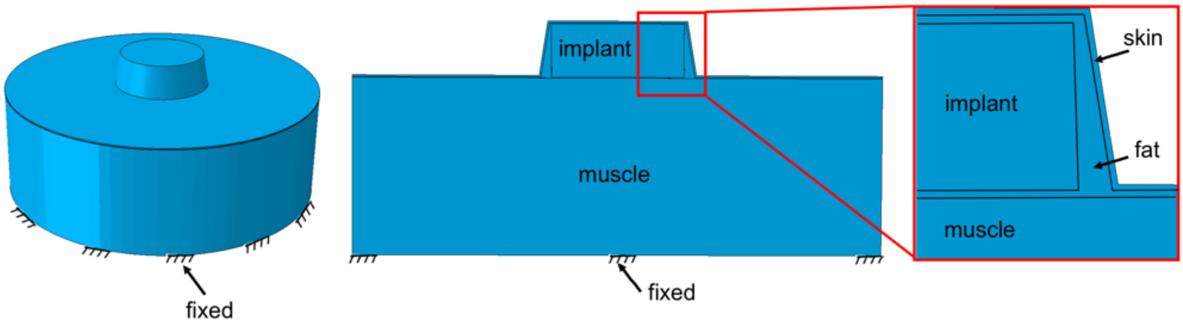

Supplementary Fig. 1 | Geometrical model used for FE modelling of biomedical implants.

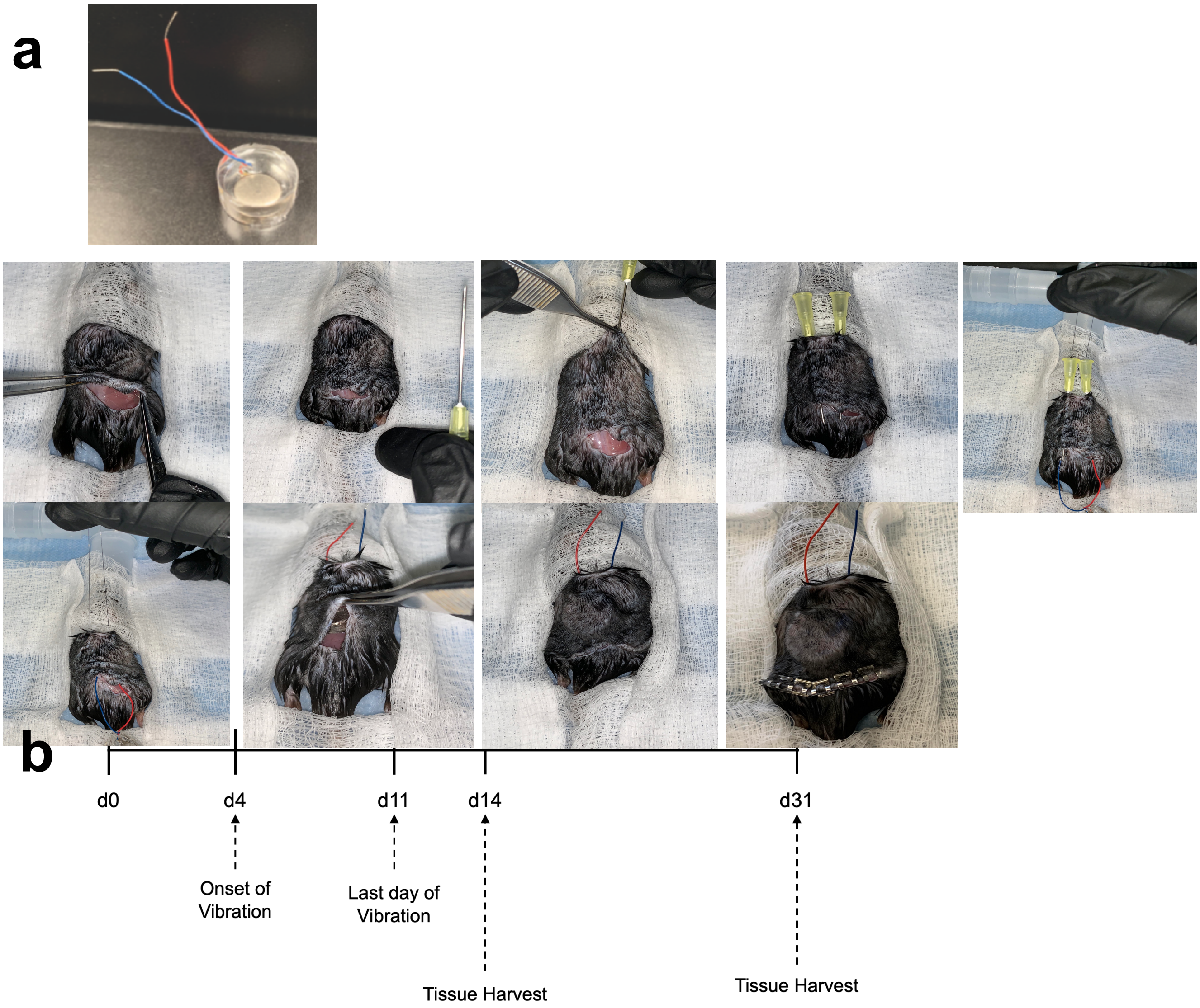

Supplementary Fig. 2 I (**a**) To induce human levels of mechanical stress in a mouse, we developed silicone implants with an encapsulated prefabricated coin motor, capable of in situ vibration. To enable in situ vibration of MSIs, the wires from the implant had to be guided through the skin, which required a novel surgical technique. After skin incision and creation of a subcutaneous pocket on the back of the mice, two 20 G cannulas were inserted into the pocket in a cranio-caudal direction. The wires were tunnelled through the pocket and guided through the skin using the cannulas and a modified Seldinger technique, enabling activation of the motor by an external battery. (**b**) MSIs were vibrated daily for 1 hour from day 4 to day 12. After iterating through combinations of vibration frequency and amplitude in our finite element model, we determined that 3V batteries with an amplitude of 1.38G and a frequency of 203 Hz would artificially increase extrinsic forces from the surrounding host tissue to generate a 100-fold increase in mechanical stress at the implant-tissue interface (24.1 kPa), similar to that surrounding human implants.

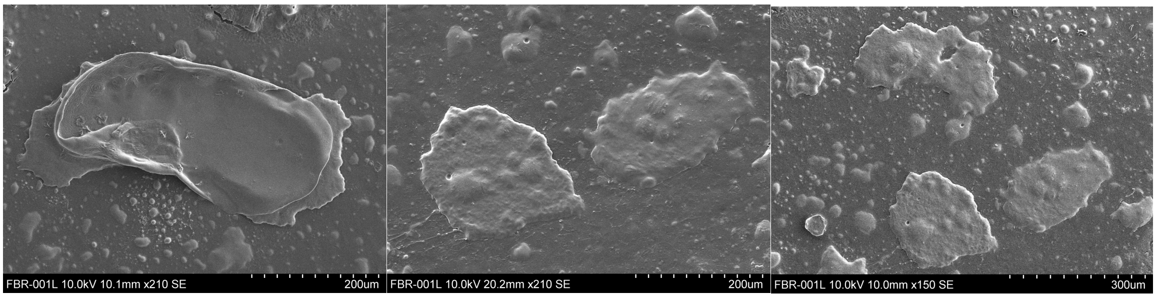

Supplementary Fig. 3 I Fusogenic macrophages were observed on the surface of human biomedical implants. Representative images are shown from similar images across n = 5 devices.
