# Supplementary material for: Allometrically scaling tissue forces drive pathological foreign-body responses to implants via Rac2-activated myeloid cells
Source: Nat Biomed Eng. 2023 Sep 25;7(11):1419–36. doi: 10.1038/s41551-023-01091-5 (PMC10651488; doi:10.1038/s41551-023-01091-5)
Supplement: Supplementary file 2 — Reporting Summary [file 41551_2023_1091_MOESM2_ESM.pdf]

nature portfolio

Corresponding author(s): Geoffrey C. Gurtner, Kellen Chen, Dharshan Sivaraj

Last updated by author(s): Aug 1, 2023

Reporting Summary

Nature Portfolio wishes to improve the reproducibility of the work that we publish. This form provides structure for consistency and transparency in reporting. For further information on Nature Portfolio policies, see our Editorial Policies and the Editorial Policy Checklist.

Statistics

For all statistical analyses, confirm that the following items are present in the figure legend, table legend, main text, or Methods section.

n/a	Confirmed	
☐	☒	The exact sample size (*n*) for each experimental group/condition, given as a discrete number and unit of measurement
☐	☒	A statement on whether measurements were taken from distinct samples or whether the same sample was measured repeatedly
☐	☒	The statistical test(s) used AND whether they are one- or two-sided<br>*Only common tests should be described solely by name; describe more complex techniques in the Methods section.*
☒	☐	A description of all covariates tested
☐	☒	A description of any assumptions or corrections, such as tests of normality and adjustment for multiple comparisons
☐	☒	A full description of the statistical parameters including central tendency (e.g. means) or other basic estimates (e.g. regression coefficient) AND variation (e.g. standard deviation) or associated estimates of uncertainty (e.g. confidence intervals)
☐	☒	For null hypothesis testing, the test statistic (e.g. F, t, r) with confidence intervals, effect sizes, degrees of freedom and P value noted<br>*Give P values as exact values whenever suitable.*
☒	☐	For Bayesian analysis, information on the choice of priors and Markov chain Monte Carlo settings
☒	☐	For hierarchical and complex designs, identification of the appropriate level for tests and full reporting of outcomes
☒	☐	Estimates of effect sizes (e.g. Cohen's *d*, Pearson's *r*), indicating how they were calculated

Our web collection on statistics for biologists contains articles on many of the points above.

Software and code

Policy information about availability of computer code

Data collection	R package Seurat (version 3.1.1) and Cell Ranger software (10X Genomics; version 3.1) were used for the single-cell analyses.
Data analysis	R package Seurat (version 3.1.1) and Cell Ranger software (10X Genomics; version 3.1) were used for the single-cell analyses.

For manuscripts utilizing custom algorithms or software that are central to the research but not yet described in published literature, software must be made available to editors and reviewers. We strongly encourage code deposition in a community repository (e.g. GitHub). See the Nature Portfolio guidelines for submitting code & software for further information.

Data

Policy information about availability of data

All manuscripts must include a data availability statement. This statement should provide the following information, where applicable:

- Accession codes, unique identifiers, or web links for publicly available datasets
- A description of any restrictions on data availability
- For clinical datasets or third party data, please ensure that the statement adheres to our policy

The main data supporting the results in this study are available within the paper and its Supplementary Information. The sc-RNA-seq data are available from the NCBI Gene Expression Omnibus via the Series accession number GSE227908 (https://www.ncbi.nlm.nih.gov/geo/query/acc.cgi?acc=GSE227908). Source data for the figures are provided with this paper. The raw and analysed datasets generated during the study are available from the corresponding authors on reasonable request.

Human research participants

Policy information about studies involving human research participants and Sex and Gender in Research.

Reporting on sex and gender	A de-identified tissue bank was used. No patient recruitment was carried out.
Population characteristics	For Fig. 1, we employed a large tissue bank for human breast-implant-capsule tissues, located at the University of Regensburg in Regensburg, Germany, which consists of over 550 unique breast tissue samples. As relatively few patients with Baker I capsules undergo revisionary surgery, our overall sample size was limited by this group. We were able to identify 9 samples of Baker I capsules in our biobank, and this determined the sample size for this study (n=10 for Baker I samples, n = 10 for Baker IV samples). The patients were of comparable ages: i) 40.6+/- 3.89 years at the time of implantation in Baker I and 35.8+/- 4.40 years in Baker IV, and ii) 50.3+/- 3.04 years at the time of explantation in Baker I and 51.0+/- 4.0 years in Baker IV. The patients had silicone breast implants placed for augmentation for a mean of 10.67+/- 2.79 years in Baker I and 15.23+/- 4.47 years in Baker IV. None of the patients previously had cancer.<br><br>For Fig. 2, explanted biomedical devices (breast tissue expanders, neurostimulator batteries and pacemaker) and the surrounding scar tissue were collected for this study and analysed. Informed consent was obtained from each patient, in accordance with the Institutional Review Board at Stanford University (IRB #41066).
Recruitment	No patient recruitment was carried out.
Ethics oversight	Patient samples from Stanford University Hospitals were collected under the approved protocol IRB# 41066. Approval for using the tissue bank at the University of Regensburg was given by the local ethic committee in Regensburg (Reference No.: 15-101-0024).

Note that full information on the approval of the study protocol must also be provided in the manuscript.

Field-specific reporting

Please select the one below that is the best fit for your research. If you are not sure, read the appropriate sections before making your selection.

☒ Life sciences ☐ Behavioural & social sciences ☐ Ecological, evolutionary & environmental sciences

For a reference copy of the document with all sections, see nature.com/documents/nr-reporting-summary-flat.pdf

Life sciences study design

All studies must disclose on these points even when the disclosure is negative.

Sample size	For the animal experiments, at least 5 mice per group were used. These sample sizes were sufficient to detect a statistically significant difference, and were chosen on the basis of prior preliminary experiments.
Data exclusions	No data were excluded.
Replication	To verify the reproducibility of our findings, we performed each major experiment on multiple animals to generate biological replicates. Each experiment was performed at least five independent times. All attempts at replication were successful.
Randomization	Animals were randomly assigned to each experimental group through blind selection.
Blinding	It was not possible to perform the experiments blinded, owing to only one group receiving mechanical stimulation. For image analyses, we used computer algorithms to quantify the metrics (aSMA staining, Herovici's staining, CODEX staining, and others) to analyse the images in an unbiased manner. For scRNA-seq, the cells were processed by a core facility (SFGF) blinded to treatment allocation; for the analysis, the data were normalized and processed according to the Seurat package in an unbiased manner.

Reporting for specific materials, systems and methods

We require information from authors about some types of materials, experimental systems and methods used in many studies. Here, indicate whether each material, system or method listed is relevant to your study. If you are not sure if a list item applies to your research, read the appropriate section before selecting a response.

Materials & experimental systems

n/a	Involved in the study	
☐	☒	Antibodies
☒	☐	Eukaryotic cell lines
☒	☐	Palaeontology and archaeology
☐	☒	Animals and other organisms
☒	☐	Clinical data
☒	☐	Dual use research of concern

Methods

n/a	Involved in the study	
☒	☐	ChIP-seq
☒	☐	Flow cytometry
☒	☐	MRI-based neuroimaging

Antibodies

Antibodies used	anti-A-SMA (1:200 dilution; Abeam ab5694), Rac2 (1:200 dilution; Fischer Scientific, DF6273), Alexa Fluor 488 secondary antibody (1:400 dilution; Thermo Fisher Scientific A32731), Mif (1:50 dilution; Abeam), Ccl4 (1:50 dilution; Lifespan Biosciences), Cxcl2 (1:50 dilution; Bio-rad), Pdgfra (1:50 dilution, Abeam), Adgrel (1:50 dilution; Abeam), Ms4al (1:50 dilution; Abeam), Coll (1:50 dilution; Abeam), Argl (1:50 dilution; Abeam), CD18 (1:50 dilution; Abeam), CD16 (1:50 dilution; Abeam), CD45 (1:50 dilution; Akoya Biosciences), CD90.2 (1:100 dilution; Akoya Biosciences), CD19 (1:50 dilution; Akoya Bioscienes), H2-A (1:100 dilution, Akoya Biosciences), CD3 (1:50 dilution, Akoya Biosciences). Antibody supplier name and clone name are listed in the additional supplementary files.
Validation	All antibodies used were validated by the suppliers. They were used at the optimized conditions according to the manufacturer's recommendations. Additional validation information and relevant publications are available on the manufacturers' websites.

Animals and other research organisms

Policy information about studies involving animals; ARRIVE guidelines recommended for reporting animal research, and Sex and Gender in Research

Laboratory animals	C57/BL6 mice, female, 6–8 weeks old, and BL6.Rac2 KO mice, female, 6–8 weeks old.
Wild animals	The study did not involve wild animals.
Reporting on sex	Not applicable.
Field-collected samples	The study did not involve samples collected from the field.
Ethics oversight	All animal work was conducted in accordance with the Administrative Panel on Laboratory Animal Care protocols (APLAC #12080 and 28410) approved by Stanford University.

Note that full information on the approval of the study protocol must also be provided in the manuscript.
